# Emergency department crowding: A systematic review of causes, consequences and solutions

**DOI:** 10.1371/journal.pone.0203316

**Published:** 2018-08-30

**Authors:** Claire Morley, Maria Unwin, Gregory M. Peterson, Jim Stankovich, Leigh Kinsman

**Affiliations:** 1 School of Health Sciences, College of Health and Medicine, University of Tasmania, Hobart, Tasmania, Australia; 2 Tasmanian Health Service–North, Launceston, Tasmania, Australia; 3 School of Medicine, College of Health and Medicine, University of Tasmania, Hobart, Tasmania, Australia; 4 Department of Neurosciences, Central Clinical School, Monash University, Melbourne, Australia; Mayo Clinic, UNITED STATES

## Abstract

**Background:**

Emergency department crowding is a major global healthcare issue. There is much debate as to the causes of the phenomenon, leading to difficulties in developing successful, targeted solutions.

**Aim:**

The aim of this systematic review was to critically analyse and summarise the findings of peer-reviewed research studies investigating the causes and consequences of, and solutions to, emergency department crowding.

**Method:**

The Preferred Reporting Items for Systematic Reviews and Meta-Analyses (PRISMA) guidelines were followed. A structured search of four databases (Medline, CINAHL, EMBASE and Web of Science) was undertaken to identify peer-reviewed research publications aimed at investigating the causes or consequences of, or solutions to, emergency department crowding, published between January 2000 and June 2018. Two reviewers used validated critical appraisal tools to independently assess the quality of the studies. The study protocol was registered with the International prospective register of systematic reviews (PROSPERO 2017: CRD42017073439).

**Results:**

From 4,131 identified studies and 162 full text reviews, 102 studies met the inclusion criteria. The majority were retrospective cohort studies, with the greatest proportion (51%) trialling or modelling potential solutions to emergency department crowding. Fourteen studies examined causes and 40 investigated consequences. Two studies looked at both causes and consequences, and two investigated causes and solutions.

**Conclusions:**

The negative consequences of ED crowding are well established, including poorer patient outcomes and the inability of staff to adhere to guideline-recommended treatment. This review identified a mismatch between causes and solutions. The majority of identified causes related to the number and type of people attending ED and timely discharge from ED, while reported solutions focused on efficient patient flow within the ED. Solutions aimed at the introduction of whole-of-system initiatives to meet timed patient disposition targets, as well as extended hours of primary care, demonstrated promising outcomes. While the review identified increased presentations by the elderly with complex and chronic conditions as an emerging and widespread driver of crowding, more research is required to isolate the precise local factors leading to ED crowding, with system-wide solutions tailored to address identified causes.

## Introduction

Emergency Department (ED) crowding has been described as both a patient safety issue and a worldwide public health problem [1]. While many countries, including Ireland [2], Canada [3], and Australia [4], report significant and unsustainable increases in ED presentations, a growing number of studies have found that these increases cannot be explained by population growth alone [4–6]. Crowding in the ED can occur due to the volume of patients waiting to be seen (input), delays in assessing or treating patients already in the ED (throughput), or impediments to patients leaving the ED once their treatment has been completed (output) [7]. Consequently, there are likely to be many different causes of crowding, depending on when and where in the patient journey the crowding occurs. Therefore, if the international crisis [8] of ED crowding is to be solved, it is crucial that interventions designed to resolve the problem are tailored to address identified causes.

Recognising that crowding had become a major barrier to patients receiving timely ED care, Asplin and colleagues [7], in 2003, issued a ‘call to arms’ to researchers and policy makers to focus their efforts on alleviating the problem. Many answered the call, and there now exists considerable published research addressing the ED crowding agenda. Despite this, and perhaps due to the relative lack of published studies investigating the causes of crowding, many myths seem to persist as to the drivers of the problem [9, 10], thereby making the implementation of successful, sustainable solutions difficult. A systematic and critical review of the available evidence can aid researchers, clinicians and managers to make decisions regarding the best course of action [11].

The most recent comprehensive synthesis of the literature, that we identified, investigating the causes, effects and solutions to ED crowding, was undertaken ten years ago (2008) [8]. With the fast changing pace of research in the emergency medicine arena, it was anticipated that in the intervening years there would have been many developments as regards identifying both causes and consequences of ED crowding, as well as the implementation of successful solutions. The aim of this review was to expand on and provide an updated critical analysis of the findings of peer-reviewed research studies exploring the causes or consequences of, or solutions to, ED crowding.

## Method

### Definition of crowding

There is currently no consensus on the correct tool or unit of measurement to define ED crowding [12], with one systematic review identifying 71 unique measures currently in use [13]. We therefore elected to include papers that had used any of the most commonly accepted metrics. These included: ED length of stay (EDLOS), rates of ‘left without being seen’ (LWBS) or did not wait (DNW), hours of ambulance bypass/diversion, hours of access block/boarding hours, proportion of presentations meeting nationally mandated, timed patient disposition targets (e.g. the Australian National Emergency Access Target (NEAT), the UK 4-hour target or the NZ Shorter-stays-in-emergency-departments target), Emergency Department Work Index (EDWIN) score, National Emergency Department Overcrowding Scale (NEDOCS) and ED census. Some studies used more than one of these measures as the dependent variable.

### Search strategy

The Preferred Reporting Items for Systematic Reviews and Meta-Analyses (PRISMA) guidelines were followed (S1 Table) [11]. A search was performed on four electronic databases: Medline, CINAHL, EMBASE and Web of Science. Search terms used were: ‘emergency department’, ‘accident and emergency’, ‘ED’, ‘emergency service’ “AND” ‘crowding’, ‘overcrowding’, ‘utilisation’, ‘congestion’ “AND” “OR” ‘consequences’, ‘outcomes’, ‘harm’, ‘negative impact’, ‘mortality’, ‘causes’ ‘strategies’, ‘solutions’, ‘interventions’. All research published in the English language between January 2000 and June 2018 was eligible for inclusion. There was no restriction on types of studies, with quantitative, qualitative and mixed-methods studies all eligible for inclusion. Studies had to satisfy the following inclusion criteria to be considered: full text original research articles, published in peer-reviewed journals, investigating the causes and/or consequences of, and/or solutions to, crowding in general EDs. As research suggests that crowding may have different effects in paediatric populations compared to adults [14], studies undertaken in paediatric EDs were excluded. Full details of the search strategy are available in supplementary material (S1 File).

### Study selection, assessment and data extraction

One reviewer (CM) reviewed the titles and abstracts to identify relevant articles. Two reviewers (CM and MU) independently reviewed the full text articles to determine which of the studies met all of the inclusion criteria. Where consensus could not be reached by discussion, a third reviewer (LK) acted as adjudicator until unanimity was achieved. Two reviewers (CM and MU) used the Scottish Integrated Guidelines Network (SIGN) critical appraisal tools [15] to assess the quality of the studies. Four reviewers worked in two pairs (MU and GP, LK and JS), using a standardised form, to extract data from the included studies. Extracted data included study design, setting and population, sample size, primary and secondary outcomes, and whether consequences affected staff, patients or the system, and causes and solutions were related to input, throughput or output factors. Disagreements were resolved by discussion until a consensus was reached, with the fifth reviewer (CM) available to act as arbitrator, if required. Details of the protocol for this systematic review were registered on PROSPERO [16] (S2 File).

## Results

**Fig 1:**
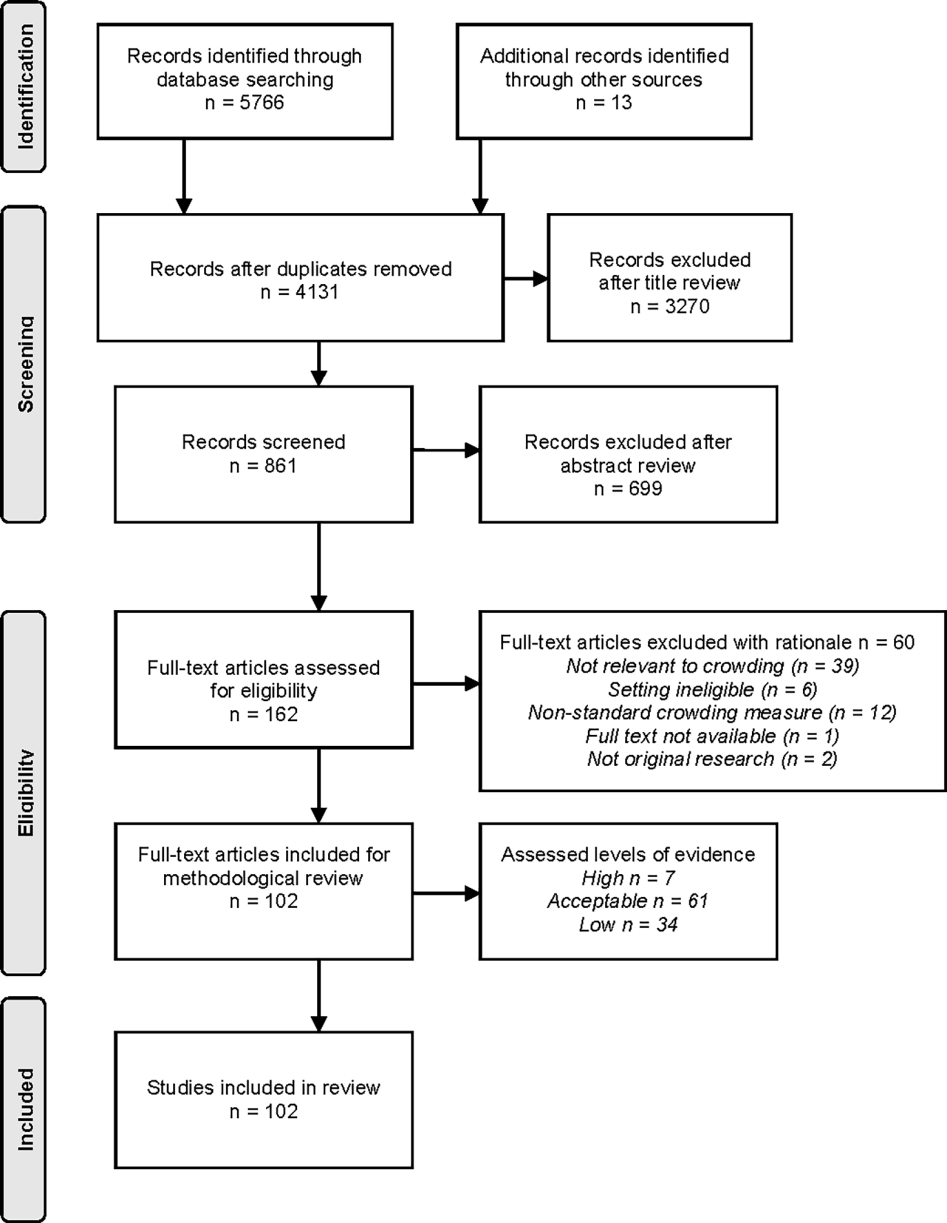
Preferred reporting items for systematic reviews.

The database search returned 5,766 articles. Thirteen additional articles were added after searching the reference lists from identified studies, leaving a total of 4,131 articles after duplicates were removed. After the initial review of titles and abstracts, 162 full text articles were retrieved for full review, with 102 of these satisfying all of the inclusion criteria, and therefore included in the final review (Fig 1).

### Study characteristics

The majority of studies were quantitative (95%) and retrospective in nature (87%), with eight prospective studies included, four each for studies investigating consequences [17–20] or solutions [21–24]. Four randomised control trials evaluating potential solutions were included [25–28], with the remaining studies being mixed-methods or statistical modelling. The majority of studies were from the USA (47%), Australia (18%) and Canada (9%), with 72% of studies having been published in the previous ten years (2009–2018). The largest proportion of studies addressed either the solutions to (51%) or consequences of (39%) ED crowding (Tables 1 and 2). Only 14 included studies (14%) investigated potential causes (Table 3). Two studies looked at both causes and consequences [29, 30], and two studies investigated causes and potential solutions [20, 31].

**Table 1:** Studies investigating potential solutions to reduce ED crowding (*n* = 52).

Author / Country /year	Design	Aim/s	Sample	Summary of intervention	Primary outcome measure/s	Level of evidence	Summary of findings
**Anantharaman / Singapore / 2008** [32]	Retrospective cohort	To review the effects of 4 social interventions on ED utilisation		1. Three public education campaigns on proper use of ED 2. Financial disincentives for ED attendance 3. Redirection of non-emergencies from the ED 4. Provision of alternative clinics for those redirected and patients with minor complaints	Average non-emergency attendance	Low	1. Smaller reductions in non-emergency attendances post each campaign 2. Decrease in non-emergency attendances increased as ED fee increased 3. Number of patients redirected declined over time. Scheme ceased due to adverse public relations incidents 4. Decrease in non-emergency attendances seen with evening clinics, but time cost to ED showed no substantial benefit. Walk-in clinics had no impact on ED attendances
**Arain / UK / 2015** [33]	Retrospective cohort and survey	To determine the impact of a GP-led WIC on the demand for ED care.	Minor attendances at 1 x Paediatric ED, 1 x Adult ED and 1 X MIU, 1 year pre and 1 year post opening of WIC 488 surveys competed	Opening of a GP-led WIC, 8:00–21:00 7 days a week	Minor attendances at 1 x Paediatric ED, 1 x Adult ED and 1 X MIU (Quant analysis) Attendances at the WIC by ‘GP-Type’ cases (Survey)	Acceptable	Significant 8.3% reduction in adult daytime GP-type attendances.
**Arya / USA / 2013** [34]	Retrospective chart review	To determine the effect of a split-level ESI 3 flow model on LOS for all discharged patients.	20,215 pre 20,653 post	‘Splitting’ of patients with ESI 3 into low and high-variability	LOS for discharged patients.	Acceptable	Significant 5.9% reduction in LOS for all patients.
**Asha / Australia / 2014** [35]	Pre-post, retrospective cohort	To determine if an emergency journey coordinator (EJC) improved NEAT compliance through resolving delays in patient processing	23,848 pre 20,884 post	Additional senior nursing role (EJC) in ED 7/7 from 14:30 to 23:00hrs. Conducted continuous rounds, focussed on patients approaching 2–3 hrs in ED, to identify delays and resolve issues to facilitate departure within 4 hrs	Proportion of patients meeting NEAT. ED occupancy. Ambulance transfer of care times. LWBS rates.	Acceptable	Significant 4.9% increase in patients meeting NEAT targets. Significant decrease of 2 patients in median ED occupancy. Non-clinically significant 56 second increase in ambulance transfer of care
**Barrett / USA / 2012** [36]	Pre-post, retrospective, cohort	To assess the impact of a bed management strategy on boarding time of admitted patients in the ED	10,967 ED presentations	Implementation of new positions to ensure timely identification and allocation of inpatient beds. Improved communication around discharge and bed availability. Education for all clinical staff re new bed management policy.	EDLOS. Time from decision to admit until transfer to inpatient bed. LWBS rates. Hrs of ambulance bypass. Hold hrs (time >1 hr in ED post admission decision).	Low	21% reduction in mean EDLOS (admitted patients) 52% reduction in boarding time. 0.7% reduction in LWBS. 11% reduction in hrs of ambulance bypass. 61% reduction in hold hrs.
**Begaz / USA/ 2017** [27]	RCT	To assess the impact of initiating diagnostic tests from the ED waiting room for patients with abdominal pain on EDLOS	848 intervention 811 control	Stable patients (usually triage cat 3) with a chief complaint of abdominal pain randomised to either undergo diagnostic testing while in the waiting room or no testing until assigned an ED bed, following a rapid medical assessment on arrival	Time in an ED bed EDLOS LWBS rate	High	Significant 32 min reduction in mean time in an ED bed Significant 44 min reduction in mean EDLOS
**Buckley / Australia / 2010** [37]	Retrospective time series analysis	To assess the impact of an after-hours GP (AH GP) clinic on the number of daily low-urgency presentations to ED	345,465 ED presentations	Opening of a user-pays AH GP clinic in a large regional centre with one ED	Daily ED presentations	Acceptable	Significant reduction of 7.04 patients per day (ATS 4&5) or 8.2% reduction in total presentations Daily increase of 1.36 patients (ATS 1,2 & 3) or 1.6% in total presentations
**Burke / Australia / 2017** [38]	Prospective observational	To assess the impact of a new model of care on EDLOS	35,428 intervention 35,623 Control	Combines clinical streaming, team-based assessment and senior consultation	EDLOS NEAT compliance LWBS rate	High	Significant reduction in mean EDLOS Significant increase in proportion of patients meeting NEAT targets Significant reduction in LWBS rate
**Burley / USA / 2007** [39]	Retrospective cohort	To assess whether quality improvement initiatives can improve flow for ED admitted patients	6 months pre, 6 months post	Consensus from key stakeholders that admitted patients not remain in ED ED patients given priority for inpatient beds Nurse handover faxed rather than telephoned Transportation staff placed in ED with priority given to admitted patients Two-tiered response to capacity limitations	Median time from bed request to assignment Median time from bed assignment to disposition EDLOS for admitted patients	Low	Significant reduction in median time from bed request to assignment in 3 of 6 months Significant reduction in median time from bed assignment to disposition in all months Significant reduction in median EDLOS in 5 of 6 months
**Burström / Finland / 2016** [40]	Pre-post, retrospective, cohort	To assess the impact of Physician led triage on efficiency and quality in the ED	20,023 pre 23,765 post	Senior physician and nurse triage all newly arrived patients. Next a team of junior physician, I x RN and 1 x nursing assistant care for patient following a detailed protocol to preform standardised work	Multiple time measures LWBS Unscheduled returns (24 and 74 hr) Mortality (7 and 30 day)	Low	Significant decreases in: EDLOS LWBS rates Unscheduled returns Mortality within 7 and 30 days of first visit
**Cha / Korea / 2015** [41]	Retrospective cohort	To determine the long-term effects of an independent capacity protocol (ICP) on ED crowding metrics	271,519 ED presentations over 6 years, 3 years pre, 3 years post	ICP converted ED into temporary, nonspecific ward. ED physicians assisted by specialists in determining disposition. When condition allowed, patients transferred to surrounding community hospitals.	EDLOS	Low	Significant decrease in EDLOS
**Chang / USA / 2018** [24]	Mixed Method	To identify strategies among high-preforming, low-preforming, and high preforming improving hospitals to reduce ED crowding		No intervention. Interview data from 60 key leaders in 4 high-performing (top 5%), 4 low-performing (bottom 5%), and 4 improving hospitals		Low	No specific strategies identified. Identified 4 organisational domains associated with high performance hospitals; executive leadership involvement, hospital-wide coordinated strategies, data-driven management and performance accountability
**Copeland / Canada / 2015** [42]	Pre-post, retrospective, cohort	To determine if ED fast-track (FT) is an efficient strategy to reduce wait times in a single physician coverage ED	7,432 ED visits	Open from 09:00–21:00hrs. 5 acute beds plus some chairs allocated to FT. Specially trained triage nurses allocated patients to either acute care or FT. Once a number of FT patients together, physician assessed and treated sequentially.	Wait time LOS	Acceptable	Significant 6 min reduction in medium wait time Significant 3.6% increase in patients meeting Canadian standard time guidelines
**Dolton / UK / 2016** [43]	Retrospective, case control	To evaluate the impact of a pilot of 7-day opening of GP practices on ED attendances	4 pilot GP practices 30 ‘control’ practices	4 geographically dispersed GP clinics opened 7 days a week. Advertised in local area and at the local ED	ED attendance	Acceptable	Significant 9.9% drop in total ED attendances Significant 17.9% drop in weekend ED attendances
**Douma / USA / 2016** [28]	RCT	To evaluate the effect of 6 nurse-initiated protocols on ED crowding	67 control 76 intervention	6 updated protocols for nurse-initiated treatment commenced. Training provided to 30 nursing staff on protocol use.	Time to diagnostic test Time to treatment EDLOS	Low	Significant 186 min reduction in time to analgesic administration Significant 79 min reduction in time to troponin measurement Significant reduction in EDLOS for 3 of 6 protocols
**^Estey / Canada / 2003** [31]	Exploratory field study	To describe the perceptions of health care professionals on potential solutions to ED crowding	Seven focus groups representing all 7 EDs in the region.	Suggestions from focus groups, no intervention		Low	Increased test turnaround-time (TAT). Better ED staffing. Faster response from admitting teams. Holding unit for admitted patients More inpatient beds 24hr outpatient appointments.
**Fulbrook / Australia / 2017** [44]	Non-randomised controlled trial	To assess the effect of a nurse navigator role on NEAT performance	9,822 intervention 9,951 control	Nurse navigator worked 12:30–20:30 on a week-on, week-off basis for 20 weeks.	NEAT compliance EDLOS	Acceptable	Significant increase in proportion of patients meeting NEAT targets Significant reduction in mean EDLOS
**Han / USA / 2008** [45]	Pre-post, retrospective, cohort	To determine the impact of physician triage on ED crowding measures	8, 569 ED visits pre 8,569 ED visits post	After nurse triage, a dedicated physician initiated diagnostics and treatments of patients in the waiting room, 7/7 between 13:00–21:00hrs	EDLOS LWBS rates Ambulance diversion hrs	Acceptable	Significant 14 min reduction in EDLOS for discharged patients Significant 2% reduction in LWBS rates Reduction in ambulance diversion hrs
**Holroyd / USA / 2007** [25]	RCT	To evaluate the implementation of triage liaison physician (TLP) shifts on ED crowding	136 shifts: 2,831 ED presentations (intervention) 133 shifts: 2,887 presentations (control)	3 x 2 week blocks where shifts randomly allocated to TLP shifts versus not (11:00–20:00hrs) TLP mitigated factors impeding throughput including: supported/assisted triage nurses, evaluated ambulance patients, initiated diagnostic studies	EDLOS LWBS rates	High	Significant 36 min decrease in EDLOS LWBS rates decreased significantly from 6.6 to 5.4%.
**Howell / USA / 2008** [46]	Pre-post, retrospective, cohort	To measure the impact of an ‘active bed management’ intervention on EDLOS and ambulance diversion hrs	17,573 ED visits pre 16,148 ED visits post	Dedicated physician role, working in 12 hr shifts, 24/7. Physician freed from all other clinical duties. Assessed real time bed availability and made collaborative triage decisions re optimal clinical setting for patient’s requiring admission. New bed director position who could call in extra staff and admit patients outside of speciality area.	Admitted and discharged EDLOS	Acceptable	EDLOS for admitted patients reduced by 98 min, with no change for discharged patients Reduction in ambulance diversion hrs
**Imperto / USA / 2012** [47]	Pre-post, retrospective, cohort	To determine if physician-in-triage (PIT) improves ED patient flow	17,631 patients	After nurse triage, PIT assessed and ordered diagnostics and treatments as required. Tasks performed by an RN and technician assigned to PIT.	Time to physician evaluation Median LOS Time to disposition decision LWBS rate	Acceptable	Significant reductions in: Median time to physician Median EDLOS Hrs on ambulance bypass
**Jang / USA / 2013** [26]	RCT	To compare EDLOS between patients assigned to metabolic Point-of-care testing (POCT) versus central laboratory testing	10,244 patients	Patients randomised to either POCT or central laboratory testing	EDLOS	High	Reduced median EDLOS by 20 min in patients assigned to POCT
**Jarvis / UK / 2014** [21]	Prospective, observational, cohort study	To compare the impact of an emergency department intervention team (EDIT) with a traditional nurse triage model on EDLOS	3,835 control 787 intervention	All ED patients assessed by EDIT Nurse history, observations and administration of initial treatments, compilation and execution of an investigation plan. All discharged patients thoroughly examined by consultant. POCT utilised as appropriate. Non-discharged patients transferred to central cubicle area for traditional care	‘Time to ED ready’ (i.e. time from registration to time all ED care complete). Time from arrival to first contact with clinical staff. Time from arrival to start of assessment by member of clinical staff.	High	Significant 53 min decrease in median time to ED ready Significant 8 min decrease in median time to assessment by member of clinical staff
**Jones / NZ / 2017** [48]	Retrospective cohort	To assess for changes in clinically relevant outcomes after the introduction of a national target for EDLOS	5,793,767 ED presentations 2,082,374 elective admissions to 18 of 20 potential district health boards	Nationally mandated that 95% of ED presentations would be admitted, discharged or transferred within 6 hrs of arrival. Wide variety of process, staffing and structural changes implemented at different hospitals	EDLOS IPLOS ED representations ≤ 48 hrs Readmissions ≤ 30 days Access block	Acceptable	Significant reduction of ^#^0.29 days in median IPLOS Significant reduction of ^#^1.1 hrs in median EDLOS No change in ED representations ≤ 48 hrs Significant ^#^1% increase in readmissions ≤ 30 days Significant ^#^27% reduction in access block ^#^Determined *a priori* to be of clinical significance
**Kelen / USA / 2001** [22]	Prospective, pre-post, observational	To determine the impact of an inpatient, ED-managed acute care unit (ACU) on ED overcrowding	10,871 ED presentations, 1,587 patients in the ACU (14.4% of ED census)	Opening of a 14-bed monitored unit, located at a distance remote to the ED, within the hospital. Staffed by ED personnel. Designed to accept ED patients who required observation or management for >4 hrs.	LWBS rates. Hrs of ambulance diversion.	Acceptable	Significant decrease in LWBS rates. Significant decrease in hrs of ambulance diversion.
**Kim / Korea / 2012** [49]	Retrospective cohort	To evaluate the effects of a short text message reminder to decision makers who delay assessing patients in the ED on EDLOS	1,693 consulted patients pre 1,642 consulted patients post	2-4-8 SMS project When no decision on patient disposition entered on computer 2 hrs post referral, SMS reminder sent to resident. Same at 4 hr mark. Admissions delayed 8 hrs, SMS sent to relevant faculty/admissions office	EDLOS Consultation time Disposition time Boarding time	Low	Significant 36 min reduction in median EDLOS for admitted patients No effect on consultation time Significant decreases in disposition and boarding time
**Lauks / Holland / 2016** [50]	Pre-post, retrospective, cohort	To assess the impact of implementing medical team evaluation (MTE) in the ED	47,743 ED visits	Physician teamed with a triage nurse, 7/7, between 09:00–22:00hrs. Physician initiated diagnostics and treatments and discharged ESI 5 patients.	Door-to-doctor time EDLOS	Acceptable	Significant 30 min decrease in median door-to-doctor time Significant 15 min increase in median EDLOS
**Lee / Taiwan / 2017** [51]	Retrospective cohort	To assess the impact of high turnover ‘ED utility beds’ on ED crowding	70,515 control 69,706 intervention	14 beds for ED patient use only with strict regulations to govern occupancy. Restriction of 48-hr limit for each patient	EDLOS LWBS rates	Acceptable	Significant 1.7 hr decrease in mean EDLOS for all admitted non-trauma patients No change in EDLOS for discharged patients No change in rates of LWBS
**Lee-Lewandrowski / USA / 2003** [52]	Pre-post, retrospective, cohort	To investigate the impact of a POCT satellite laboratory in the ED	369 patients	Clinicians had option of central laboratory or POCT for urinalysis, pregnancy testing, cardiac markers and glucose	Test TAT EDLOS	Low	87% reduction in test TAT Significant 41 min decrease in EDLOS for combined patients having 3 tests (excluding glucose) No significant decrease for patients having single test EDLOS for patients who did not receive POCT increased by non-significant 11 min
**Lee-Lewandrowski / USA / 2009** [53]	Pre-post, retrospective, cohort	To evaluate the impact of implementing rapid D-dimer testing in an ED satellite laboratory	252 patients pre 211 patients post	24 hr satellite laboratory in the ED had ability to undertake rapid D-dimer testing	Test TAT EDLOS	Low	79% decrease in test TAT Significant 1.32 hr decrease in mean EDLOS for patients who received D-dimer testing
**Mason / UK / 2011** [54]	Retrospective data analysis	To evaluate the effect of the mandated ED care intervals in England	735,588 ED visits from 15 hospitals over 4 years. Mix of high, middle and low performing	Nationally mandated 4 hr target for patient disposition for 98% of ED presentations. Specific interventions not detailed but hospitals expected to adopt a whole-systems approach	EDLOS Time to first ED clinician review	Acceptable	Proportion leaving ED within 4 hrs increased from 83.9 to 96.3% Median EDLOS for admitted patients decreased by 25 min
**McGrath / USA / 2015** [55]	Retrospective cohort	To evaluate the impact of a flexible care area (FCA) on ED throughput measures	417 days over 2 years when FCA was operational	3 roomed area staffed by ED physician, RN and ED technician from 16:00–23:00hrs. Prioritised moderate acuity to expedite ordering of diagnostics	EDLOS LWBS rate	Low	Significant decrease in EDLOS for some ESI categories Significant reduction in rates of LWBS
**McHugh / USA / 2013** [56]	Retrospective, cross-sectional	To evaluate the efforts of five hospitals (a-e) that introduced various interventions to reduce ED crowding		a. PIT b. Faxed report from ED to admitting unit and bed coordinator c. Adoption of ESI triage scale, bedside registration and staff resourcing for ED fast-track area d. More efficient intake process for non-urgent patients e. Improved process to request specialist consults	EDLOS LWBS rates	Low	a. Significant reduction in EDLOS for mid-acuity patients (target group) b. Significant reduction in LWBS rates c. Significant reduction in EDLOS d. Significant reduction in EDLOS e. Increase in EDLOS
**Mumma / USA / 2014** [57]	Retrospective cohort	To determine the effects of ED expansion on ED crowding	42,896 pre 48,358 post	ED expanded from 33 to 53 beds No substantial changes to physician staffing or nurse/technician to patient ratios	LWBS rates Daily boarding hrs	Low	No change in LWBS rates Significant increase in boarding hrs from 160 hrs per day to 180 hrs per day
**Nagree / Australia / 2004** [58]	Retrospective, cohort	To model the capacity of after-hours GP services to reduce low acuity presentations (LAPs) to metropolitan EDs	183, 424 ATS 3–5 patients	No intervention. Modelling the impact of AH GP services	Excess LAPs	Acceptable	After-hours GP services for LAPs are unlikely to significantly reduce total ED attendances or costs
**Ngo / Australia / 2018** [59]	Retrospective cohort	To assess the impact of the Western Australia (WA) 4 hr target on ED functioning and patient outcomes	3,214,802 ED presentations across 5 hospitals (2002–2013)	Implementation of a 4 hr rule (NEAT) whereby 90% of ED patients in the state of WA were to be admitted, discharged or transferred within 4 hrs of arrival	Access block ED occupancy rate ED re-attendances ≤ 1 week EDLOS	Acceptable	Significant decrease in percentage of access block at all hospitals Significant decrease in median ED occupancy at 4 of 5 hospitals Significant decrease in median EDLOS at 4 of 5 hospitals
**Partovi / USA / 2001** [60]	Retrospective, cohort	To investigate the effect of Faculty triage on EDLOS	8 intervention days 8 ‘control’ days	A faculty member was added to the triage team of 2 nurses and one emergency medicine technician. Their role included: rapid evaluation, move serious patients to main area, order diagnostics and fluids, discharge simple cases and encourage rapid registration	Nurse triage time Nurse discharge time LWBS rates	Low	Significant 82 min reduction in mean EDLOS
**Patel / USA / 2014** [61]	Pre-post, retrospective, cohort	To assess the effect of a leadership-based program to expedite hospital admissions from the ED	25 months pre 47 months post	Team of hospital leaders convened. Computerised tracking system used to monitor ED bed status in real time. Agreement to admit patients within 1 hr of decision to admit	Proportion of ED patients admitted to inpatient bed within 60 mins of bed request	Acceptable	Significant 16% increase in proportion of patients admitted within 60 mins of bed request
**Perera / Australia / 2014** [62]	Pre-post, retrospective, cohort	To assess the effect of NEAT on common crowding metrics	76,935 patients	Hospital-wide education program to increase awareness of NEAT initiative	EDLOS IPLOS Proportion of admissions meeting NEAT Mortality rates	Acceptable	Significant improvements in: EDLOS NEAT admission targets Access block Significant increase in IPLOS No change to mortality rates
**Quinn / USA / 2007** [63]	Pre-post, retrospective, cohort	To determine the impact of a rapid assessment policy (RAP) on EDLOS	10,153 pre 10,387 post	ED physicians directly admit patients to inpatient beds. Admitting physicians not required to assess patients in the ED prior to admission No requirement for all laboratory and radiological test results to be complete prior to admission	EDLOS Time on ambulance diversion. LWBS rates.	Acceptable	Significant 10 min decrease in EDLOS Significant 65% decrease in hrs of ambulance diversion
**Sharma / Australia / 2011** [64]	Statistical modelling	To model the determinants of duration of wait of ATS 2 patients in an ED and test whether diverting ATS 5 patients away from the ED, or increasing ATS 5 patients’ choice of EDs reduces ED waiting times for ATS 2 patients.	84,291 ATS 2 199,973 ATS 5	No intervention. Modelling the impact of co-located GP and choice of ED for ATS 5 patients on outcomes for ATS 2 patients	EDLOS	Low	Co-located GP significantly reduced mean wait of ATS 2 patients by 19% Increasing choice of ATS 5 patients beyond a certain number of ED options had a negative effect on duration of wait for ATS 2 patients
**Shetty / Australia / 2012** [23]	Prospective, interventional	To assess the impact of the ‘Senior Streaming Assessment Further Evaluation after Triage (SAFE-T) zone’ concept on ED performance	10,185 pre 10,713 post	Developed an assessment zone around triage to facilitate early physician review, disposition decision-making, and streaming to bypass the ED acute area	EDLOS LWBS rates	High	Significant reductions in: EDLOS for ATS 2–5 LWBS rates
**Shin / Korea / 2017** [65]	Retrospective cohort	To measure the effect of an improved speciality consultation process on EDLOS	6,967 pre 7,301 post	Between 7am and 6pm only senior emergency physicians (as opposed to emergency residents) consult internal medicine (IM) physicians re patients requiring admission. If required, the IM physician reviews the patient in the ED and organises prompt resident review for admission	EDLOS of IM patients Admission order to ED departure Overall EDLOS Discharged EDLOS	Acceptable	Significant 290 min reduction in mean EDLOS Significant 120 min decrease in mean time from admission order to ED departure No change to overall EDLOS No change to discharged EDLOS
**Singer / USA / 2008** [66]	Retrospective, cohort	To investigate the effect of a dedicated ED ‘stat’ laboratory on EDLOS	5,631 ED visits pre 5,635 ED visits post	A stat laboratory dedicated to ED patents set up within the main laboratory, staffed by dedicated personnel, 24/7	EDLOS for admitted patients	Low	Significant 21 min reduction in median EDLOS for all patients with laboratory tests performed Significant 62 min reduction in median EDLOS for admitted patients with laboratory tests performed
**Sullivan / Australia / 2014** [67]	Retrospective, pre-post, interventional	To evaluate the effect of various reforms (throughput and output) to meet the NEAT target of disposition from ED within 4 hrs	All ED presentations for the same 3-month periods in 2012 (pre), 2013 (post) and 2014 (maintenance)	Senior staff taskforce set up to provide oversight, direction and monitor NEAT compliance. Business intelligence unit set up to make reporting transparent. Compliance seen as whole-of-hospital flow problem. Major redesign of clinical processes, including bed management operations	Proportion of patients exiting ED within 4 hrs Mean transit times within the ED Inpatient mortality LWBS rates 48 hr representation rates	Acceptable	Significant increase in: Proportion of patients exiting ED within 4 hrs Mean transit times within the ED Significant decrease in: Inpatient mortality LWBS rates
**Takakuwa / USA / 2006** [68]	Retrospective, cohort	To investigate the effect of bedside registration on EDLOS	52,225 patient encounters	When beds were available, patients brought immediately back to patient care area following triage where they were registered by a clerk whilst being simultaneously assessed by medical staff	Time from triage-to-room Time from room-to-disposition	Low	Significant decrease in time from triage-to room with bedside registration for non-urgent patients
**Tenbensel / NZ / 2017** [69]	Mixed method	To assess the impact of a national 6 hr target for ED admissions on EDLOS To identify particular actions that impacted on identified reductions in EDLOS	4 hospitals covering 25% of NZ population 68 semi-structured interviews	Nationally imposed target of 95% of all ED presentations seen, treated or discharged within 6 hrs	Reported EDLOS Total EDLOS (includes time in short-stay unit) Staff perceptions of successful interventions	Acceptable	Reductions in median reported EDLOS in all hospitals Smaller reductions in median total EDLOS in all hospitals ***Results from interviews*** Hospital leadership prior to target New resources (beds and staff) Processes to improve flow within the ED and hospital wide Improved information and communication
**van der Linden / Holland / 2013** [70]	Retrospective, cohort	To investigate the effect of a flexible acute admissions unit (FAAU) on EDLOS for admitted patients and inter-hospital transfers	8,377 ED visits pre 8,931 ED visits post	Between 4pm and 8am daily at least 15 potential FAAU beds were identified across several inpatient units. During office hours, patients were transferred back to ‘home’ departments where possible. Employment of an ‘admissions coordinator’ who assessed the bed status in real time	Number of admissions transferred to other hospitals EDLOS for patients requiring ‘regular’ admission (non-specialist) EDLOS for discharged patients	Low	Significant decrease in number of patients transferred to other hospitals due to bed unavailability No change in EDLOS for patients admissible to FAAU in comparison to increased EDLOS for ‘other’ admissions
**^van der Linden / Holland / 2017** [20]	Mixed Method	To compare staff perceptions of causes and solutions of ED crowding in two EDs: one in Pakistan and one in The Netherlands	18 one-hour staff interviews 12 in Pakistan 6 in The Netherlands	Suggestions from interviews, no intervention		Low	An additional triage room More staff to reduce delays in decision to admit More efficient processes for bed management and diagnostics An acute admissions unit More effective bed coordination
**^White / USA / 2012** [71]	Pre-post, retrospective, cohort	To assess the impact of ‘Supplemented Triage and Rapid Treatment’ (START) on ED throughput	12,936 pre 14,220 post	After nurse triage, non-FT patients assessed by a physician who ordered diagnostics and identified patients whose disposition could be accelerated without further need for clinical work-up in the ED.	EDLOS LWBS rates	Acceptable	Significant decrease in: Median EDLOS LWBS rates
**Whittaker / UK / 2016** [72]	Retrospective cohort	To investigate the association between extending GP opening hrs and ED visits for minor injuries	2,945,354 ED visits	4 ‘schemes’ (each scheme serves population of 200–300,000 people) received funding to provide additional urgent and routine GP appointments between 5-9pm Mon-Fri and on both days of the weekend	Per capita (per 1,000) patient-initiated ED referrals for minor problems Total ED visits	Acceptable	Significant 26% relative reduction in patient-initiated ED referrals for minor problems in intervention practices Insignificant 3.1% relative reduction in total ED visits
**Willard / USA / 2017** [73]	Retrospective cohort	To examine the effectiveness of a Full Capacity Protocol (FCP) to reduce ED crowding	20,822 ED encounters control 22,357 ED encounters intervention	A predetermined response to specific circumstances in the hospital and ED. Additionally, can be activated by ED coordinator in response to reduced throughput. When activated, hospital leaders gather in ED to collaboratively identify and remove barriers to obtaining disposition.	LWBS rates EDLOS Ambulance diversion hrs	Acceptable	10.2% non-significant decrease in LWBS rates Significant 34 min increase in mean EDLOS Significant 92% decrease in total hrs of ambulance diversion

^Papers also looked at causes of crowding

ACU = acute care unit AH = after hours ATS = Australian triage scale ED = emergency department EDIT = emergency department intervention team EDLOS = emergency department length of stay EJC = emergency journey coordinator ESI = emergency severity index FAAU = flexible acute admissions unit FCA = flexible care area FCP = full capacity protocol FT = fast-track GP = general practitioner ICP = independent capacity protocol IM = internal medicine IPLOS = inpatient length of stay LAP = low-acuity presentation LOS = length of stay LWBS = left without being seen MIU = minor injury unit MTE = medical team evaluation NEAT = National Emergency Access Target PIT = physician in triage POCT = point-of-care test RAP = rapid assessment policy RN = registered nurse SMS = short-message-service TAT = turnaround-time TLP = triage liaison physician WIC = Walk-in centre

**Table 2:** Studies investigating potential consequences of ED crowding (*n* = 40).

Author/ Country /year	Design	Aim/s	Sample	Primary outcome measure/s	Level of evidence	Summary of findings
*Bond / Canada / 2007 [29]	Non-comparative survey	To investigate the frequency, determinants and impacts of ED crowding	158 ED Directors	Frequency, determinants and impacts of ED crowding	Low	Increased stress of clinical staff Increased wait times Provider dissatisfaction Risk of poor patient outcomes and delay in improvements in patients’ wellbeing
**Chiu / Taiwan / 2018** [74]	Retrospective cohort	To investigate the effect of crowding on clinical efficiency, diagnostic tool use and patient disposition	70,222 ED visits in 2 EDs	Time to disposition decision EDLOS Patient disposition Diagnostic interventions ordered	Acceptable	Increased odds of being admitted in times of crowding Slightly increased odds of CT scanning and laboratory testing during crowding
*Derlet / USA / 2002 [30]	Non-comparative survey	To determine the incidence, causes and effects of crowding in EDs in three US states	210 ED directors	Incidence, causes and effects of ED crowding	Low	Delayed commencement of therapy across a range of conditions leading to poor outcomes for patients
**Diercks / USA / 2007** [75]	Secondary data analysis from an observational registry	To evaluate the association between EDLOS, guideline-adherence to recommended therapies and clinical outcomes of patients presenting to the ED with non-ST-segment-elevation myocardial infarction (non-STEMI)	42,780 patients with non-STEMI	Adherence to 5 acute guideline medication recommendations (defined as receiving medications within 24 hrs) Occurrence of in hospital adverse events (death, recurrent MI)	Acceptable	Long ED stays associated with decreased use of guideline-recommend therapies and a higher risk of recurrent MI No observed increase in inpatient mortality
**Fee / USA / 2007** [76] `	Retrospective cross-sectional, chart review	To determine the association between ED volume and timing of antibiotic administration in patients admitted via the ED with community acquired pneumonia (CAP)	405 patients with CAP	Did/did not receive antibiotics within 4 hrs in relation to total ED volume. Time to antibiotics in relation to number of patients in the ED who were ultimately admitted.	Acceptable	Higher ED volume independently associated with a lower likelihood of patients with CAP receiving antibiotics within 4 hrs (OR 0.96 per additional patient). Number of patients in the ED ultimately admitted had a slightly stronger, but non-significant, effect than the number of patients ultimately discharged, on time to antibiotics (OR 0.93 Vs 0.97).
**Gaieski / USA / 2017 [77]**	Retrospective cohort	To investigate the hypothesis that ED crowding would impact negatively on the care of patients with severe sepsis or septic shock	2,913 patients with severe sepsis	Time to administration of intravenous fluids (IVF) Time to administration of antibiotics Initiation of protocolized care (Y/N) Inpatient mortality	Acceptable	ED occupancy had significant negative impact on odds of patients receiving IVF within ≤ 1 hr and antibiotics within ≤3 hrs Number of boarders in the ED had significant negative impact on the odds of receiving protocolized care No impact on inpatient mortality
**Guttmann / Canada / 2011** [3]	Retrospective cohort	To determine whether patients discharged from the ED during shifts with long waiting times are at risk for adverse events	13,934,542 patients discharged from ED	Admission to hospital or death within seven days	Acceptable	Patients presenting to EDs during shifts with long mean waiting times might be at increased risk of death and admission in subsequent 7 days, regardless of acuity on presentation
**Hwang / USA / 2006** [78]	Retrospective cohort	To evaluate the effect of ED o/c on assessment and treatment of pain in older adults with a hip fracture	158 patients	Documented pain assessment Time to pain assessment Documentation and administration of analgesic Type of analgesic administered	Low	When the ED was at >120% capacity there was a significant reduced odds of patients having their pain documented on first assessment and a longer time to pain assessment. No impact on time to administration of analgesic
**Hwang / USA / 2008** [79]	Retrospective cohort	To evaluate the association of ED crowding factors with quality of pain care	1,068 ED visits	Time to documented pain assessment Time to medications ordered and administered Type of analgesia ordered	Acceptable	ED census directly associated with significant delays in: Pain assessment Time to analgesic ordering and administration
**Jo / Korea / 2015** [80]	Retrospective cohort	To evaluate the association between ED crowding and inpatient mortality among critically ill patients admitted through the ED	1,801 critically ill patients (systolic BP<90mmHg)	Inpatient mortality	Acceptable	ED crowding associated with increased inpatient mortality
**Kulstad / USA / 2009** [81]	Retrospective cohort	To determine the association between percutaneous coronary angiogram (PCI) for patients presenting to ED with an acute myocardial infarction (AMI) and ED crowding	17 patients who underwent PCI over a 2-month period	Time to first Electro-cardiogram (ECG) Time to arrival at cardiac catheterisation lab (CCL) Time to first balloon inflation	Low	No relationship between time to ECG and time to arrival in the CCL and crowding Significant delay in time to balloon inflation during times of crowding (p = 0.008)
**Kulstad / USA / 2010** [17]	Prospective, observational	To determine the association between ED crowding and the frequency of medication errors	6,728 EDWIN scores and 283 medication errors	Correlation between the average daily EDWIN score and total number of daily medication errors detected	Low	Significant positive correlation between average daily EDWIN score and medication error frequency (p = 0.001)
**Lee/ Taiwan / 2012** [18]	Prospective, chart review	To investigate the factors related to blood culture contamination in the ED	558 patients with positive blood cultures	Rates of contaminated blood cultures in relation to ED crowding as measured by the NEDOCS	Low	ED overcrowding independently associated with contaminated blood cultures (OR 1.58, p = 0.04). Strong correlation between contamination rate and degree of ED crowding (Pearson correlation = 0.99, p = 0.001)
**Liew / Australia / 2003** [82]	Retrospective cohort	To examine the association between EDLOS and IPLOS	17,954 admissions	Mean IPLOS Excess IPLOS	Low	EDLOS is associated with excess IPLOS
**Liu / USA / 2011** [83]	Retrospective cohort	To examine the relationship between ED boarding and quality of care amongst patients admitted for chest pain, pneumonia or cellulitis	1,431 patients included	Medication delays and errors. Adverse events.	Acceptable	Boarding time associated with home medication delays (AOR 1.07 CI 1.05–1.10)
**McCarthy / USA / 2009** [84]	Retrospective cohort	To quantify the relationship between ED crowding and EDLOS	235,928 ED visits at 4 EDs	Waiting room time Treatment time Boarding time	Acceptable	Crowding delayed waiting room and boarding time but not treatment time Crowding delayed the care of ATS 2 patients at all sites
**McCusker / Canada / 2014** [85]	Retrospective cohort	To examine the association of ED occupancy with patient outcomes	677,475 patients at 42 EDs	Deaths at 30 days for both admitted and discharged patients Return ED visits for discharged patients Admission following return ED visit	Acceptable	A 10% increase in ED bed relative occupancy ratio was associated with a significant 3% increase in death
**Medley / USA / 2012** [86]	Retrospective chart review	To determine if there is an association between ED occupancy rates and violence towards ED staff	278 included cases	The presence of violent incidents	Acceptable	A significant association between crowding and violence towards staff
**Mills / USA / 2009** [87]	Secondary data analysis from a prospectively collected database	To evaluate the association between ED crowding and analgesic administration in adult ED patients with acute abdominal pain	976 patients with abdominal pain	Receipt of analgesia Delays in administration of analgesia	Acceptable	ED crowding not associated with failure to treat with analgesia Higher crowding levels in ED independently associated with significant delays in administration of analgesia
**Nippak / Canada / 2014** [88]	Retrospective cohort	To identify the relationship between EDLOS and IPLOS	4,743 ED visits	EDLOS IPLOS	Low	Positive significant correlation between EDLOS and IPLOS
**Pines / USA / 2006** [89]	Cross-sectional, data-linkage	To assess the association between ED crowding and antibiotic timing in pneumonia and PCI in AMI	Administrative data from 24 EDs	Time to antibiotic administration in patients with pneumonia Time to PCI in patients with AMI	Low	An increase in overall EDLOS associated with a significant decrease in percentage of patients receiving antibiotics within 4 hrs (p = 0.04) No association between ED crowding measured and time to PCI for patients with AMI
**Pines / USA / 2007** [90]	Retrospective cohort	To assess the impact of ED crowding on delays in antibiotic administration for patients with community acquired pneumonia (CAP)	694 patients with CAP	Time from patient triage until antibiotic administration	Acceptable	Crowding in the ED is related to delayed and non-receipt of antibiotics in patients with CAP
**Pines / USA / 2008** [91]	Retrospective cohort	To study the impact of ED crowding on ED patients with severe pain	13,758 patients	Receipt of any analgesia Delay of >1 hour from triage to receipt of analgesia Delay of >1 hour from arrival in a treatment room to receipt of analgesia	Acceptable	Increasing levels of ED crowding were significantly associated with failure to treat or delayed treatment with analgesia
**Pines / USA / 2009** [92]	Retrospective cohort	To examine whether ED crowding was associated with adverse cardiovascular outcomes in patients with chest pain syndrome	4,574 patients	The development of an adverse cardiovascular outcome that was not present on ED arrival, but that occurred during hospitalisation	Acceptable	A positive association between some measures of ED o/c and rates of adverse cardiovascular outcomes
**Pines / USA / 2010** [93]	Retrospective cohort	To study the association between ED crowding and the use of, and delays in administration of analgesia in patients with back pain	5,616 patients	Receipt of any analgesic Time to administration of analgesia	Acceptable	Higher crowding levels in the ED independently associated with significant delays in analgesia administration
**Reznek / USA / 2016** [94]	Retrospective cohort	To investigate the hypothesis that ED crowding is associated with longer door-to-imaging time (DIT) in patients with acute stroke	463 patients	DIT ≤ 25 mins (Y/N)	Acceptable	Crowding had a significant negative impact on DIT
**Richardson / Australia / 2002** [95]	Retrospective cohort	To assess the relationship between access block in the ED and IPLOS	11,906 admissions	EDLOS and IPLOS	Acceptable	Patients who experienced access block had a significant mean IPLOS 0.8 days longer than those who did not experience access block
**Richardson / Australia / 2006** [96]	Retrospective stratified cohort	To quantify any relationship between ED o/c and 10-day inpatient mortality	34,377 patients (o/c shifts) 32,231 patients (non-o/c shifts)	In-hospital death recorded within 10 days of most recent ED presentation	Acceptable	ED patients presenting in times of o/c had significantly higher 10 day in-hospital mortality than those presenting to a non-o/c ED
**Richardson / Australia / 2009** [97]	Retrospective cohort	To identify any relationship between access block and the time to definitive care of patients with fractured neck of femur.	369 cases of fractured neck of femur	Time to surgery (<24 hrs = ‘timely’) in relation to ED crowding as measured by access block occupancy (ABO) quartile	Acceptable	Significant relationship between ABO quartile at presentation and delayed surgery (p = 0.006)
**Sikka / USA / 2010** [98]	Retrospective cohort	To measure the correlation between ED occupancy rate and time to antibiotic administration for patients with pneumonia	334 patients	Initial antibiotic administration within 4 hrs of ED arrival	Acceptable	Significant negative association between time to antibiotic treatment and ED crowding, as measured by ED occupancy rate
**Singer / USA / 2011** [99]	Retrospective cohort	To explore the association between ED boarding and clinically important patient outcomes	41,256 admissions from the ED	In-hospital mortality	Acceptable	Prolonged ED boarding negatively associated with significant increase in in-hospital mortality and significant increase in IPLOS
**Sprivulis / Australia / 2006** [100]	Retrospective cohort	To examine whether high hospital occupancy and ED access block are associated with increased inpatient mortality	62,495 hospital admissions	Deaths on days 2, 7 and 30 evaluated against an overcrowding hazard scale	Acceptable	Hospital and ED o/c is associated with a 30% relative increase in mortality by Day 2 and Day 7 for patients requiring admission via ED to an inpatient bed
**Sun / USA / 2013** [101]	Retrospective cohort	To assess the association of ED crowding with subsequent outcomes in a general population	995,379 ED visits resulting in admission to 187 hospitals	Inpatient mortality	Acceptable	High ED crowding associated with: 5% greater odds of inpatient death 0.8% increase in IPLOS
**Tekwani / USA / 2013** [102]	Retrospective cohort and patient survey	To evaluate the impact of ED crowding on satisfaction of patients discharged from the ED	1,591 patient satisfaction scores over 497 8-hr shifts	Mean patient satisfactions scores Modified EDWIN score ED census Ambulance diversion rate	Low	ED crowding significantly associated with decreased patient satisfaction (p < 0.001)
**Tsai / Taiwan / 2016** [103]	Retrospective cohort	To investigate the impact of crowding and number of ED staff on efficiency of ED care processes for patient with acute stroke presenting ≤ 3 hrs of symptom onset	1,142 acute stroke patients	Door-to-assessment time (DTA) Door-to-computed tomography completion time (DTCT) Door-to-needle (DTN) time where appropriate	Low	DTA and DTCT times significantly increased in times of crowding No effect on DTN time
**van der Linden / Holland / 2016** [104]	Retrospective Chart review	To assess the impact of ED crowding on triage processes	45, 539 ED presentations	Target time to triage (mandated target time = 10 mins) Any triage score assigned	Acceptable	ED crowding associated with: significant delay in target time to triage significant number not assigned a triage score
**Verelst / Belgium / 2015** [19]	Prospective observational	To determine whether ED crowding was independently associated with in-hospital death within 10 days of ED admission	32,866 admissions	Risk-adjusted HR for in-hospital death occurring within 10 days of ED admission in crowding quartile 4 vs. occupancy quartiles 1, 2 and 3	High	No significant association between ED crowding and overall risk of mortality
**White / USA / 2013** [105]	Retrospective cohort	To investigate the effect of boarding hospital inpatients in the ED on LOS of patients discharged from the ED	179,840 discharged patients	Discharged patient LOS	Acceptable	As boarder burden increased, EDLOS for discharged patients increased by 10%
**Wickham / Sweden / 2017** [106]	Retrospective cohort	To investigate the effect of crowding on EDLOS of ten most common medical or surgical complaints	19,200 ED visits 4,456 high acuity 14,744 low acuity	Median EDLOS for 10 chief complaints, stratified by high acuity (triage scores 1&2) and low acuity (triage scores 3–5)	Acceptable	Significant 46% increase in EDLOS for high acuity patients in times of crowding, true for all complaints except ‘wound’ Significant 82% increase in EDLOS for low acuity patients in times of crowding, true for all 10 conditions studied
**Zhou / China / 2012** [107]	Retrospective cohort	To investigate whether patients boarded in the ED are subjected to increased serious complications	20,276 admitted patients	New onset of shock Need for intubation Death within 24 hrs of decision to admit	Acceptable	Positive correlation between high daily hospital occupancy and rates of shock and intubation, but not death within the initial 24 hrs post-admission request

*Papers also looked at causes of crowding

ABO = access block occupancy AMI = acute myocardial infarction AOR = adjusted odds ratio ATS = Australian triage scale BP = blood pressure CAP = community acquired pneumonia CCL = cardiac catheterisation laboratory CT = computerised tomography DIT = door-to-imaging time DTA = door-to-assessment time DTCT = door-to-computed-tomography time DTN = door-to-needle time ECG = electrocardiograph ED = emergency department EDLOS = emergency department length of stay EDWIN = Emergency Department Work Index HR = hazards ratio IPLOS = inpatient length of stay IVF = intravenous fluid NEDOCS = National Emergency Department Overcrowding Scale o/c = overcrowding/ed OR = odds ratio PCI = percutaneous coronary angiogram

**Table 3:** Studies investigating potential causes of ED crowding (*n* = 14).

Author / Country /year	Design	Aim/s	Sample	Outcome measure/s	Level of evidence Quality assessment	Summary of findings
**Aboagye-Sarfo / Australia / 2015** [108]	Population-based longitudinal study	To analyse recent trends and characteristics of ED presentations in Western Australia (WA)	All ED presentation in WA between 2007–2013	Annual number and rates of ED presentations	Acceptable	Key drivers of increased ED presentations (4.6% annually) were people with urgent and complex care needs
*Bond / Canada / 2007 [29]	Non-comparative survey study	To investigate the frequency, determinants and impacts of ED crowding	158 ED Directors	Frequency, determinants and impacts of ED crowding	Low	Access block EDLOS Increased complexity and acuity of patients Lack of access to primary care Non-urgent patients
*Derlet / USA / 2002 [30]	Non-comparative survey study	To determine the incidence, causes and effects of o/c in EDs in three US states	210 ED directors	Incident, causes and effects of ED o/c	Low	Access block Increased severity of conditions Increased ED volume
**Cowling / UK / 2013** [109]	Cross-sectional, population based	To examine the association between access to primary care and ED visits	7,856 GP practices	Number of self-referred, discharged, ED visits by the registered population of a general practice	Acceptable	Significantly less self-referred, discharged, ED visits from practices that provided timely access
**Dunn / Australia / 2003** [110]	Pre-post, retrospective, cohort	To determine if changes in hospital occupancy would affect ED occupancy and ED wait time performance	1,133 ED visits pre 2,332 ED visits post	Hospital occupancy Access block days EDLOS LWBS rate	Acceptable	Significant decrease in: Hospital occupancy Access block days EDLOS LWBS rate
**^Estey / Canada / 2003** [31]	Exploratory field study	To describe the perceptions of health care professionals regarding service pressures that result in ED overcrowding	Seven focus groups representing all 7 EDs in the region. Groups consisted of ED physicians (8), ED managers (8), and other ED staff including nursing and allied health (42).		Low	Shortage of inpatient beds Change of role of ED as ‘holding unit’ for the rest of the healthcare system Shortage of nursing staff Limited access to diagnostic services Increased numbers of high-acuity, elderly patients
**Fatovich / Australia / 2005** [111]	Retrospective data analysis	To systematically evaluate the relationship between access block, ED o/c, ambulance diversion and ED activity	259,580 ED attendances	Hrs on ambulance diversion Hrs of access block	Acceptable	Ambulance diversion and poor ED performance were related to poor inpatient flow, access block
**Forster / Canada / 2003** [112]	Retrospective data analysis	To identify the effect of hospital occupancy on EDLOS for admitted patients and patient disposition	351,385 ED visits	EDLOS Rate of daily referral from ED to specialist admitting teams	Acceptable	EDLOS significantly associated with hospital occupancy No association between hospital occupancy and decision to admit
**Kawano / Japan / 2014a** [113]	Cross-sectional, single-centre	To assess and model associations between types of ED staff and ED crowding	27,970 ED visits	Proportion of patients with a clinically significant delay EDLOS	Low	No significant negative association between presence of junior residents and clinically significant delay ***Results of modelling***: Adding 1 junior resident increased EDLOS for all patients Extra senior resident reduced EDLOS for discharged patients Extra attending physician reduced EDLOS for all patients
**Kawano / Japan / 2014c** [114]	Retrospective data analysis	To estimate the increase in EDLOS with the trend of an ageing society	15,840 ED visits	EDLOS	Acceptable	Increase in older patients vising the ED has a significant negative effect on ED o/c
**Knapman / Canada / 2010** [115]	Retrospective, cohort	To assess the impact of aged patients (≥65) in the ED on ED crowding, wait times and patient flow for non-emergent patients	223 patients	Wait time to see a physician	Low	Strong relationship between aged patients in the ED and increased wait time for non-emergent patients
**Lucas / USA / 2009** [116]	Retrospective, cohort	To determine the effect of hospital census variables on EDLOS	27,325 ED visits	EDLOS Daily ED volume Proportion of ED admissions Daily hospital census Daily census of critical care and cardiac telemetry units Daily number of scheduled surgeries	Low	Significant negative relationship between EDLOS and ICU census, cardiac telemetry census and percentage of ED patients admitted
**Moineddin / Canada / 2011** [117]	Data modelling	To assess the factors resulting in increased demand for ED services in a Canadian province	53,353 respondents to a Canadian nationwide survey exploring (among other things) health system utilisation	Number of ED visits in a year	Acceptable	Access to a primary care provider significantly reduces the odds of an ED presentation for low-severity conditions (triage categories 4&5)
**^van der Linden / Holland / 2017** [20]	Mixed method	To compare staff perceptions of causes of ED crowding in two EDs: one in Pakistan and one in The Netherlands	18 one-hour staff interviews 12 in Pakistan 6 in The Netherlands	Staff perceptions of causes and solutions to ED crowding	Low	Increase in elderly patients and patients with complex conditions Delays in triaging Wait time for diagnostic procedures Delays in decision to admit Access block

*Papers also looked at consequences of crowding

^Paper also looked at solutions to crowding

ATS = Australian triage scale CT = computerised tomography ED = emergency department EDLOS = emergency department length of stay GP = general practitioner ICU = = intensive care unit LAP = low-acuity presentation LWBS = left without being seen o/c = overcrowding/ed

### Study quality

The SIGN appraisal tools guidelines [15] recommend that all retrospective or single cohort studies receive a rating of no higher than ‘acceptable’. Consequently, the majority of the included studies (59%) were rated as being of acceptable quality. The remaining studies were rated as high (7%) and low (34%) quality. The main area of weakness was inadequate consideration of potential confounders, leading to uncertainty about claims of cause and effect. The level of statistical analysis was often basic, with confidence intervals frequently absent in the reporting of results and few multivariate analyses. Similarly, although percentage and time improvements were frequently noted, often there was no indication whether or not the improvement values were statistically significant. Two survey studies [29, 30], one focus group study [31], and two interview studies [20, 24] without confirmatory numerical data, were also included. Furthermore, with the exception of one study [19], all of the 40 studies that investigated the consequences of crowding reported negative effects. Similarly, all included studies evaluating potential solutions, with two exceptions [57, 58], reported significant improvements in measures of crowding, leading to questions about the potential for publication bias in this area of research. As regards the solution studies, in many cases it was not possible nor advisable to blind clinicians to the interventions. This makes them vulnerable to the Hawthorne effect, whereby an initiative improves outcomes as participants are aware that their practice is being observed and therefore modify their behaviour. However, for the majority of the interventions in this type of health services improvement research it could be unethical to undertake a blinded randomised control trial.

### Consequences of ED crowding

Forty of the included studies examined the consequences of ED crowding, with three of these being prospective [18, 19, 81] (Table 2). Almost all were undertaken in single EDs and reported negative consequences associated with ED crowding. The included studies investigating the consequences of ED crowding can be broadly categorised into patient, staff or system level effects (Table 4).

**Table 4:** Studies reporting consequences of ED crowding.

**Patient Effects** Poor patient outcomes e.g. for patients with chest pain [29, 30, 75, 92, 107] Increased mortality [3, 80, 85, 96, 99–101] Delayed assessment and care [29, 30, 76–79, 81, 83, 84, 87, 89–91, 93, 94, 98, 103, 104], including surgery [97] Increased IPLOS [82, 88, 95, 99, 101] Risk of readmission [3, 74] Reduced patient satisfaction [102] Exposure to error [17, 18]
**Staff Effects** Non-adherence to best practice guidelines [18, 75–79, 90, 91, 93, 94, 98, 103, 104] Increased staff stress [29] Increased violence towards staff [29, 86]
**System Effects** Increased IPLOS [82, 88, 95, 99, 101] Increased EDLOS [29, 84, 105, 106]

IPLOS = inpatient length of stay EDLOS = emergency department length of stay

### Patient

Effects on patients included delays in being assessed and receiving required care [29, 30, 76–79, 81, 83, 84, 87, 90, 91, 93, 94, 97, 98, 103, 104], increased frequency of exposure to error [18], including medication errors [17], reduced patient satisfaction [102], increased inpatient length of stay (IPLOS) [82, 88, 95, 99, 101] and poorer outcomes [29, 30, 75, 92, 107]; the latter included increased inpatient mortality [3, 80, 85, 96, 99–101].

#### Delayed assessment and treatment

A significant delay in time to balloon inflation for patients experiencing an acute myocardial infarction and transferred to the cardiac catheterisation laboratory (CCL) from the ED during times of crowding, was identified in one American retrospective cohort study [81]. Similarly, delays in undergoing surgery for patients presenting to crowded EDs with a fractured neck of femur, were identified in one Australian study [97]. A number of studies investigated the effects of crowding on time to medication administration in the ED. Findings were predominantly adverse, with crowding associated with delays in time to receive analgesic [79, 87, 91, 93] and antibiotic therapy [76, 77, 89, 90, 98], as well as delays in patients receiving their usual prescribed or ‘home’ medications [83]. Two studies reported negative impacts of crowding on timely care for patients with acute stroke [94, 103]. One study reported significant delays in triage times, with a significant number of patients not assigned any triage score in times of crowding [104].

#### Exposure to error

One American prospective observational study identified an increased frequency of medication errors, including the administration of incorrect and contraindicated medications, during times of crowding [17]. As well as delays in receiving medication, three studies reported an association between crowding and total failure to administer required analgesics or antibiotics [87, 90, 91]. ED crowding was independently associated with increased rates of blood culture contamination in one Taiwanese study, with the rate of contamination strongly correlated with the degree of crowding [18].

#### Increased IPLOS

All of the five studies examining the relationship between ED crowding and IPLOS reported a positive association [82, 88, 95, 99, 101]. One Australian study compared the effect of access block on the IPLOS of 11,906 admitted patients, and reported a mean increased IPLOS of 0.8 days in patients who experienced access block [95]. Richardson’s study highlighted that the access block effect on IPLOS was relatively independent of illness severity or diagnosis, but was greatest in patients admitted in the out-of-hours period [95]. Similarly, Sun and colleagues reported a 0.8% increase in IPLOS for patients admitted via an ED which was experiencing crowding, defined by this group as the top quartile of the daily number of ambulance diversion hours [101].

#### Increased inpatient mortality

Although the majority of papers investigating the effects of ED crowding on inpatient mortality reported that as crowding worsened mortality increased, three studies found no relationship [19, 75, 77]. Two of the studies were focussed on specific groups of patients, namely patients presenting with non-ST-segment-elevation myocardial infarction (non-STEMI) [75] and patients with severe sepsis [77]. The third study, undertaken in a tertiary teaching hospital in Belgium, was the only prospective study included in this review that specifically investigated inpatient mortality [19]. Verelst and colleagues measured the outcomes of 32,866 adult patients admitted via the ED over a two-year period. They divided crowding into four quartiles, based on the ratio of the total number of ED patients to the total number of treatment bays, with quartile four considered as ED crowding. After adjusting for severity of illness they reported no association between ED crowding and risk of inpatient mortality at 10 days [19].

Conversely, the seven retrospective studies that investigated the effect of ED crowding on inpatient mortality all reported that mortality increased as crowding worsened [3, 80, 85, 96, 99–101]. The varying results can be partially explained by differences in study designs, making it difficult to compare findings between studies. There were wide-ranging differences in measures of crowding, with daily hours of ambulance diversion [101], boarding time for admitted patients [99], mean ED occupancy [96], EDLOS [3] and relative ED occupancy [80, 85] variously applied as proxy measures of crowding. Similarly, there were differences in study populations, with most studies including all adult admitted patients [96, 99–101]. However, one study included only critically ill admitted patients [80], another included admitted and discharged patients [85], and another study considered only the outcomes for patients discharged from the ED [3]. However, Verelst et al. justified their finding of no association between ED crowding and increased risk of inpatient mortality as being due to their large sample size, controlling for multiple confounders and their use of a validated measure of crowding, in this case ED occupancy rate [19].

### Staff

Identified negative effects on staff included increased stress [29], increased exposure to violence [29, 86], and non-adherence to best practice guidelines during times of ED overcrowding [18, 75–79, 89–91, 93, 94, 98, 103, 104]. Arguably, the latter could also be positioned with consequences for patients, but here we use it in the context of staff being unable to properly undertake their roles during times of increased crowding.

#### Increased stress and violence

In a Canadian survey study of 158 ED directors, increased stress among nurses was the most commonly perceived major or serious impact of ED crowding [29]. Staff stress was identified by more participants as an issue than increased wait times or poor patient outcomes. Increased physician stress was also identified as being driven by crowding [29]. A significant association between ED crowding and violence towards staff was reported in one study involving a retrospective chart review [86]. Physical violence was the most frequently documented type, with violence directed towards staff the majority of the time [86].

#### Adherence to guidelines

Poor adherence to approved guidelines was reported to be a consequence of ED crowding in 13 studies [18, 75–79, 89–91, 93, 94, 98, 103]. Increased time to assessment of pain and/or delays in administration of analgesics were found to be positively associated with ED crowding in all four studies investigating this issue [78, 79, 91, 93]. Similarly, of six studies investigating the effects of crowding on time to antibiotic therapy initiation, five identified a positive association between delayed time to administration and ED crowding [76, 77, 89, 90, 98]. One American study, involving the analysis of data from a voluntary registry tracking guideline adherence, found that patients with non-STEMI who boarded for long periods of time in the ED were less likely to receive guideline-recommended therapies and were at higher risk for repeat MI [75].

### System

System-level consequences identified were those that led to ‘bottle-necks’ in the system, namely increases in length of stay (LOS), both within the ED itself (EDLOS) [29, 84, 105, 106] and also for those patients admitted to the hospital (IPLOS) [82, 88, 95, 99, 101]. Again, these could also be viewed as consequences for patients.

#### Increased EDLOS

The three studies that investigated the impact of crowding on EDLOS reported that EDLOS increased with increased crowding. An American, multi-site, retrospective cohort study investigated the effect of crowding on the EDLOS of 226,534 ED presentations at four sites over 12 months [84]. McCarthy and colleagues reported that (i) the number of patients in the waiting room had the greatest impact on time spent in the waiting room, (ii) the number of boarders in the ED was the most consistent factor associated with delays in ED care and (iii) more positively, ED crowding had little effect on time to treatment [84]. While studying only the outcomes in terms of EDLOS of discharged patients, White et al. reported a 10% increase in EDLOS for patients who presented during times of crowding, defined by this group as the top quartile of boarder burden [105]. One Swedish study reported significant increases in median EDLOS for both high and low acuity patients presenting with one of the ten principal medical or surgical complaints during times of crowding [106].

#### Increased IPLOS

As reported under patient effects previously, all of the studies examining the relationship between ED crowding and IPLOS reported a positive association [82, 88, 95, 99, 101]. It should be noted that in the literature this is generally taken to mean that ED crowding leads to increases in IPLOS; however, as is the case with all observational studies, this type of research can only identify an association between EDLOS and IPLOS rather than identifying with any certainty a causative relationship in either direction. For instance, long IPLOS could reduce the availability of beds for patients in ED waiting to be admitted, thereby worsening ED crowding. This limitation is identified in the majority, but not all, of the observational studies included in this review.

### Causes of ED crowding

Fourteen included studies investigated potential causes of ED crowding. The majority were retrospective cohort or data analysis studies, with four qualitative explorations [20, 29–31], and two data modelling studies [113, 117] (Table 3). Using the conceptual model of ED crowding developed by Asplin et al. [7], which divides ED crowding into three interdependent components, the studies that focussed on the causes of crowding can be broadly categorised as identifying input, throughput or output causes (Table 5).

**Table 5:** Studies identifying causes of ED crowding.

**Input** Presentations with more urgent and complex care needs [20, 29–31, 108] Increase in presentations by the elderly [20, 31, 114, 115] High volume of low-acuity presentations [29, 117] Access to primary care [29, 109, 117] Limited access to diagnostic services in community [31]
**Throughput** ED nursing staff shortages [30, 31] Presence of junior medical staff in ED [113] Delays in receiving test results and delayed disposition decisions [20]
**Output** Access block [20, 29–31, 110–112] ICU and cardiac telemetry census [116]

ICU = Intensive Care Unit

### Input

Causes of crowding related to the input phase of the ED process suggested increases in types of presentations, including those with urgent and complex needs [20, 29–31, 108], low-acuity presentations (LAPs) [29, 117], and presentations by the elderly [20, 31, 114, 115], as the main drivers. Access to appropriate care outside of the ED was identified as an issue in four studies [29, 31, 109, 117].

#### Types of presentations

Increased complexity and acuity of patients were perceived to be a cause of ED crowding by 54% of respondents in one American survey study [29]. A similar finding was replicated in an interview study comparing perceived causes of crowding in the Netherlands and Pakistan [20]. Similarly, a 4.6% annual average increase in ED presentations over a six-year period was attributed to increases in presentations of people with urgent and complex care needs, in a population-based longitudinal study in one Australian state [108]. Aboagye-Sarfo and colleagues reported significant increases in presentations allocated Australian Triage Score (ATS) 2 and 3 (high acuity), as well as increases in patients requiring admission, and found that a greater proportion of patients admitted over the six-year period were aged 65 years and older [108]. Increased ED presentations by the elderly, as a factor contributing to crowding was a finding of two Canadian studies, one a retrospective cohort study [115] and the other exploratory field work involving seven focus groups with key ED staff [31]. Likewise, a Japanese study that undertook a cross-sectional analysis of all adult ED presentations at one ED concluded that older people in the ED had a significant negative impact on ED crowding [114]. Kawano et al. reported that crowding worsened as the mean age of patients in the ED increased [114].

Conversely, two studies reported that increased presentations by patients with LAPs was a driver of ED crowding [29, 117]. One was the result of survey research with 158 ED directors [29], while the other was the result of statistical modelling undertaken using the results of a large number of surveys exploring Canadian health system utilisation [117]. Moineddin et al. reported that improved access to primary care could significantly reduce the odds of ED presentations for patients with LAPs [117].

#### Access to other forms of care

Poor access to primary care was identified as a cause of ED crowding in four studies [29, 31, 109, 117]. A large UK study that used a cross-sectional, population-based design to investigate whether timely access to GP care led to fewer self-referred ED visits, reported an association. The model developed by this group predicted 10.2% fewer self-referred ED visits for those GP practices ranked in the top quintile for access, with patients able to secure a GP appointment within two days less likely to self-refer to the ED with low acuity conditions [109]. Similarly, a Canadian study concluded that having access to a primary care provider had the potential to reduce non-urgent ED visits (patients allocated triage categories 4 or 5) by 40% [117].

#### Throughput

ED nursing staff shortages as a cause of ED crowding was highlighted in exploratory fieldwork undertaken with 158 ED directors in Canada [31], and in one American study that surveyed 210 ED directors [30]. Adding one junior doctor to a shift increased the EDLOS for discharged patients by one minute, while having no statistically significant effect on EDLOS for admitted patients, in one Japanese study that modelled the effect of additional staff on EDLOS [113]. One interview study that compared the views of ED staff in the Netherlands and Pakistan on causes of crowding identified delays in receiving laboratory test results and delays in patient disposition decisions as issues in both countries [20]. These low quality, predominantly opinion-based studies, were the only included publications to suggest a throughput cause for crowding.

### Output

All studies that reported on output factors as a cause of ED crowding concluded that access block, that is, the inability to transfer a patient out of the ED to an inpatient bed once their ED treatment has been completed, was the major contributor [20, 29–31, 110–112, 116].

#### Access block

Two studies analysed both ED and inpatient datasets to understand the relationship between hospital occupancy, access block and ED crowding [111, 112]. The Canadian study reported a significant relationship between ED crowding and hospital occupancy, with a 10% increase in hospital occupancy leading to an 18 minute increase in average EDLOS [112]. The Australian group found a linear relationship between ED occupancy during periods of hospital access block and total ED occupancy, with a similar relationship noted between access block and ambulance diversion and EDLOS, two other commonly used indicators of crowding [111]. An American multi-site, retrospective cohort study reported a significant positive relationship between mean EDLOS for both intensive care and telemetry bed census, but did not find a significant relationship between ED crowding and total hospital census [116]. Lucas et al. acknowledged that EDLOS is likely to be impacted by total hospital census in times of high occupancy (>90%) but as the majority of their study was undertaken on days of occupancy <90%, the study would have been unable to detect this association [116].

One small Australian study used a novel approach to investigate the effect of access block on crowding. A short period (13 days) of industrial action led to the cancellation of all elective surgery and therefore to significant improvements in bed availability for ED admitted patients [110]. Dunn compared ED performance during the time of increased bed access with a 13-day period prior to and a 13-day period after the industrial action. When there was no elective surgery and an associated reduction in hospital occupancy, there were significant reductions in access block days, EDLOS for patients allocated triage categories 2–5 (ATS 1 excluded from analysis), and patients who did not wait for treatment [110]. Similarly, results of survey research with ED directors [29, 30] and multi-site, focus group research with key ED staff [31], highlighted lack of inpatient bed availability as one of the main perceived causes of ED crowding.

### Solutions to ED crowding

Fifty-two of the included studies trialled, modelled or suggested potential solutions to ED crowding. The majority were retrospective, with four RCTs [25–28], one statistical modelling [64], and four prospective interventional studies [21–23, 38] (Table 1). Again, Asplin’s [7] conceptual model can be used to categorise the studies that investigated potential solutions to crowding in the ED (Table 6).

**Table 6:** Studied and suggested solutions to ED crowding.

**Input** GP-led walk-in centres / Co-located GP [32, 33, 64] Extended GP opening hours [37, 43, 58, 72] Choice of ED [64] Social interventions including; education campaigns, financial disincentives, redirection [32]
**Throughput** Split ESI 3 on presentation [34] Earlier physician assessment [21, 23, 38, 50, 63, 65, 67, 71], including physician-led/supported triage [25, 40, 45, 47, 56, 60] Fast-track / flexible care area [42, 55, 56] Shorter turnaround-times for laboratory tests [26, 27, 52, 53, 66] ED nurse flow coordinator [35, 44, 69] Bedside registration [56, 68] Nurse initiated protocols [28] Earlier inpatient consultation [49] Increased ED bed numbers [57, 69] Increased ED staff [69]
**Output** Active bed management [20, 36, 39, 46] Leadership program/Support [39, 61, 67] Implementation of nationally mandated, timed patient disposition targets [48, 54, 59, 62, 67, 69] ED staff direct admit rights [63, 67] Admitting team prioritise ED admissions [67] Alternative admission policies [22, 41, 69, 70, 73] Increased inpatient beds and staff [69]

GP = general practitioner ESI = Emergency Severity Index

### Input

Input factors focused on improved access to other forms of care, such as GP-led walk-in centres (WIC) [32, 33], a co-located GP within or near EDs [64], extended GP opening hours [37, 43, 58, 72] or providing a choice of ED [64]. Results of a number of social interventions were trialled over a 12 year period in one study from Singapore [32].

#### Co-located GPs and walk-in centres

The effect of a co-located GP on duration of wait for triage category 2 (high acuity) patients in the ED was modelled in one Australian study [64]. Sharma and Inder reported a 19% lower wait time for category 2 patients in EDs with a co-located GP, when compared to EDs without a GP [64]. The impact of a GP-led WIC on demand for ED care was the focus of one UK study [33]. This group used linear modelling to estimate the effect of the WIC on daytime GP-type attendances to other urgent care services in the area. A significant reduction of 8.3% in GP-type presentations to adult EDs was reported [33]. Opening of a WIC in Singapore was found to have no effect on ED presentations as the authors reported that the WICs attracted their own clientele who were unlikely to have attended the ED [32].

#### Increased GP opening hours

Another UK group evaluated the impact of a pilot 7-day opening of GP practices in central London [43]. Their analysis highlighted a significant, 17.9% reduction in weekend ED attendances by patients registered with practices involved in the pilot program. Dolton and Pathania also reported both a 19% fall in admissions among the elderly and a 29% reduction in elderly cases arriving by ambulance [43]. Similarly, another UK study that investigated the effect of later opening hours and 7-day opening of GP practices reported a 26% relative reduction in patients registered with the intervention practices self-referring to EDs with minor problems [72]. The opening of an after-hours (AH) GP located in a large regional Australian town, serviced by one ED and with limited AH services, resulted in a significant 8.2% daily decrease in total ED presentations of patients allocated ATS 4 and 5 (low acuity) [37]. Buckley et al. also reported an unexplained increase in ED presentations of those allocated ATS 1–3 (high acuity), of 1.36 per day, but that the opening of the AH service led to a ‘gradual permanent change’ in ED presentations [37].

Conversely, another Australian study that modelled the effect of AHs GPs on LAPs to six EDs in Perth, Western Australia, concluded that providing AHs GP LAP services was unlikely to reduce ED attendance, as LAPs were an ‘inexpensive but constant part of ED workload’ [58].

#### Social interventions

A study that reported on a number of social intervention trialled in Singapore over a 12 –year period reported mixed results. Public education campaigns were found to be effective initially but presentations reverted to pre-campaign levels some months after the end of each campaign [32]. Implementation of financial disincentives for non-emergency presentations began to reduce presentations once the fee exceeded the fees charged by primary health care clinics [32]. Redirection of non-emergencies to alternate facilities was successful initially, but was discontinued due to adverse public relations incidents [32].

### Throughput

The majority of studies (60%) that reported on potential solutions to ED crowding focussed on expediting patients’ throughput within the ED. These potential solutions mainly concentrated on ‘front-ending’ care earlier in the patient journey by providing earlier physician assessment [21, 23, 38, 50, 63, 65, 67, 71], including physician-led triage [25, 40, 45, 47, 60]. Dividing patients by level of acuity on arrival has also been successful in increasing throughput times, whether by opening a fact-track or flexible care area for lower acuity presenters [42, 55], or dividing patients within the same triage code [34]. Other throughput interventions included reducing the turnaround-time of laboratory tests [26, 27, 52, 53, 66], the introduction of an ED nurse flow coordinator [35, 44, 69], increasing medical and nursing staff numbers in the ED [69], bedside registration immediately following triage [68], nurse initiated protocols [28], strategies to ensure earlier review by admitting teams [49] and increasing bed numbers in the ED [57, 69].

#### Early physician assessment

Eight included papers investigated the effects of early physician assessment on measures of ED crowding [21, 23, 38, 50, 63, 65, 67, 71]. Seven of these studies reported significant decreases in EDLOS [21, 23, 38, 63, 65, 67, 71], while four reported significant decreases in numbers of patients who either LWBS or DNW [23, 38, 67, 71]. One Australian group introduced a suite of interventions to improve throughput and output within their large tertiary ED, which had previously been named as the worst preforming ED in Australia in terms of its NEAT ‘4-hour-rule’ compliance [67]. Sullivan et al. also reported significant reductions in inpatient mortality rates between baseline and the post-reform period.

Conversely, when one Dutch urban ED initiated Medical Team Evaluation as a means of improving ‘front-end operations’ through a host of initiatives, including team triage and a quick registration process, results showed a significant increase in EDLOS for patients in triage categories 2–4, regardless of discharge destination [50]. Lauks and colleagues attributed this rise to the increase in orders for diagnostic radiology during the intervention period [50].

Five groups investigated the effect of a physician in triage (PIT) model on common ED crowding metrics [25, 40, 45, 47, 60]. Although the interventions were slightly different, all involved a senior physician triaging patients early in their arrival to the ED. All reported a significant reduction in EDLOS post implementation; however, one found this decrease to apply only for patients who were subsequently discharged [45]. Han and colleagues did report an increase in boarding time for admitted patients during the intervention period, a potential reason for the intervention having little effect on EDLOS for admitted patients [45]. Only one study reported a significant decrease in patients who left without being seen [40], and two studies reported significant reductions in the number of hours on ambulance bypass during the intervention period [45, 47]. Significant decreases in both 7-day and 30-day mortality post ED visit were also reported by Burström et al. after the introduction of a PIT scheme [40].

#### Fast-track and flexible-care areas

Fast-track [42] or use of a flexible-care area [55] to improve flow within the ED were reported in two papers. Both of these studies reported significant reductions in EDLOS for triage category 4 (low acuity) patients only. As the majority of patients diverted to these areas were triaged as category 4, it is not surprising that the intervention had the greatest effect in this patient group. The fast-track group also reported significant improvements in meeting national standards for wait times for patients triaged as category 4 [42]. Similarly, an American group that geographically separated triage category 3 patients with low variability (that is, with conditions likely to follow a standardised work flow), in order to fast-track these patients through the department, reported significant decreases in EDLOS for all category 3 and 4 discharged patients [34]. Arya and colleagues attributed the decreased LOS for higher variability category 3 patients to the decreased throughput of patients through the urgent area of the ED, thereby reducing the workload of staff in this area [34].

#### Reducing laboratory test turnaround-times

Reducing the time taken to turnaround laboratory tests as a means of reducing EDLOS was investigated in four studies. Three studies reported on the use of point-of-care testing (POCT) in the ED versus central laboratory pathology testing [26, 52, 53], while one employed dedicated laboratory technicians within the central laboratory who were available 24/7 to undertake all laboratory testing for the ED [66]. All four studies reported significant reductions in EDLOS attributed to the interventions, although one noted that the reduction in EDLOS was only significant if patients had all three available tests performed [52]. One American group undertook a RCT to assess the impact of earlier initiation of diagnostic tests whilst triage category 3 patients with abdominal pain were in the waiting room [27]. Begaz and colleagues reported a significant 44 minute reduced mean EDLOS for patients randomised to the intervention versus the control arm of the trial [27].

#### ED nurse flow coordinator

The introduction of a senior nurse (emergency journey coordinator), focussed on identifying and resolving delays for patients who had been in the ED for 2–3 hours, led to a 4.9% significant increase in the number of patients meeting NEAT targets in one Australian ED [35]. Similarly, a nurse navigator role trialled as part of a non-RCT reported significant increases in the proportion of patients meeting NEAT time and reductions in mean EDLOS during days when the trial was operational [44]. A NZ group, who investigated the impact of nationally mandated times for patient disposition at four hospitals, reported the introduction of nurse flow coordinators at all four institutions as one of many interventions introduced to successfully reduce crowding [69].

#### Other

Bedside registration immediately following triage, occurring concurrently with physician evaluation, resulted in a significant decrease in time from triage to treatment room allocation for non-urgent patients, in one American before-after intervention study [68]. However, after an initial significant reduction in room-to-disposition time, this improvement was not sustained to 12 months after the intervention [68]. Three of six nurse-initiated protocols were reported to significantly reduce mean EDLOS in one American study [28]. A Korean study that used short text message reminders when ED patients waited more than two and more than four hours for inpatient consultations resulted in a significant 36 minute reduction in median EDLOS for admitted patients [49]. The expansion of the ED from 33 to 53 beds, with no changes to staffing ratios, resulted in a significant 20 hours per day increase in ED boarding in one American study [57]. Conversely, in one NZ study, provision of extra ED beds in three out of the four hospitals studied, as well as the provision of additional ED nursing and medical staff, resulted in a decreased median EDLOS [69].

### Output

Solutions looking at output factors exclusively focused on getting admitted patients out of the ED in a timely manner once their ED assessment and treatment was complete, that is, reducing access block. Suggested and trialled strategies included more active bed management [20, 36, 39, 46], leadership support to expedite hospital admissions from the ED [24, 39, 69] including leadership programs [61, 67], and implementation of nationally mandated timed disposition targets [48, 59, 62, 67, 69], which have included; giving ED staff admitting rights [63, 67], ensuring admitting teams prioritise patients waiting in the ED during times of high ED census [67], and increasing inpatient bed capacity [69]. Alternative admission units, including an ED-managed, acute care unit [22] and flexible acute admission units [51, 69, 70], have also been trialled. Implementation of an independent or full capacity program to provide alternative options for admission in times of crowding has been trialled in two studies [41, 73].

#### Bed management

An active bed management strategy to alleviate ED crowding was evaluated by one American study [46]. The initiative resulted in a 98 minute average reduction in EDLOS for admitted patients, as well as a reduction in the number of hours the hospital was on alert, in this case limiting the types of patients that could be transported by ambulance to the ED [46]. The intervention strategy involved introducing a bed manager who assessed bed availability in real time and who could triage and admit patients to inpatient beds, and a bed director who could call on other resources, including extra staff or admitting medical patients to non-medical beds, to avoid the hospital being put on alert [46]. Similarly, an intervention that included the implementation of a position to ensure timely identification and allocation of beds, coupled with improved communication and education for staff around a new bed management strategy, resulted in a mean 21% decrease in EDLOS for admitted patients, and a 52% reduction in mean boarding time in one American ED [36]. When ED patients were given priority over inpatient beds, as one of a number of quality improvement initiatives to reduce crowding in one American study, there was a significant reduction in median time from bed assignment to disposition and significant reductions in median EDLOS [39].

#### Leadership programs and leadership support

One American hospital convened hospital leaders and ED staff to work collaboratively to expedite hospital admissions from the ED [61]. This group introduced a computerised tracking system to ensure the ability for real time tracking of ED admit wait times. The group agreed to measurable goals in terms of the time between the decision to admit and final transfer to an inpatient bed. Patel and colleagues reported a significant 16% increase in patients transferred to an inpatient bed within 60 minutes of the decision to admit [61]. The group also reported significant decreases in boarding time, patients who LWBS and hours of ambulance diversion [61]. An Australian group also convened a taskforce with senior executive sponsorship to provide oversight and direction for initiatives to improve hospital admission targets [67]. Results of this initiative have been discussed under throughput solutions above and access targets, below. An American study that endeavoured to identify the different strategies used by high preforming, low preforming and improving hospitals, in relation to their levels of ED crowding found that no specific interventions were related to performance level [24]. They did, however, report that four organisational domains were associated with high preforming hospitals, one of which was executive leadership involvement [24]. Tenbensel and colleagues reported that leadership involvement in influencing cultural change was a key factor in implementing hospital-wide initiatives to meet mandated, timed admission targets in NZ [69].

#### Introduction of nationally mandated, timed, patient disposition targets

Six studies have recently reported on the effect of timed patient disposition targets on commonly reported ED crowding measures [48, 54, 59, 62, 67, 69]. One Australian study reported hospital-wide education to increase awareness of NEAT in the six months prior to its implementation as the only intervention [62]. Perera et al. reported a significant increase in the number of patients leaving the ED within the guideline recommended 4-hours, post-NEAT implementation, which was sustained in their second evaluation period, one-year post-implementation [62]. A significant reduction in access block was also reported. However, this group also found a significant increase in IPLOS and in the numbers of inter-unit transfers within 48 hours of admission. They attributed this to the possibility of ‘rushed referrals’ by ED staff in an effort to meet NEAT targets [62].

Conversely, Sullivan et al. report on a plethora of reforms introduced at their large, tertiary referral hospital [67]. These included reforms both within the ED itself, as well as hospital-wide interventions. Many of these initiatives were aimed at reducing access block in the ED, such as: ED staff able to organise direct admission for stable patients, clear limits on response times to ED referrals by inpatient teams, and improved processes for timely discharge of inpatients [67]. As discussed under throughput solutions above, this group reported significant decreases in EDLOS and inpatient mortality [67]. The only negative outcome reported by this group was a small, but statistically significant, increase in re-presentations to the ED within 48 hours, which was seen by the researchers to be clinically insignificant [67].

Ngo and colleagues reported on a longitudinal analysis of the effect of NEAT on five hospitals in Western Australia, without giving the specifics of interventions introduced at each hospital prior to NEAT implementation [59]. Similar to the above studies, they reported significant reductions in percentage of access block hours in all five hospitals and significant decreases in median EDLOS, primarily for high acuity (ATS 1–3) patients, at three out of the five hospitals [59]. The UK study did not give the specifics of interventions but stated that a whole-system approach was expected to be adopted to achieve the target [54]. Mason et al. reported a 29% reduction in the proportion of patients who remained in the ED after four hours as well as a 25% reduction in unadjusted median EDLOS for admitted patients [54].

The NZ studies also reported reductions in median EDLOS post target implementation [48, 69]. One study reported on the outcomes in relation to when they had the biggest impact and their success in relation to the increased use of short-stay units (SSU) [69]. Tenbensel and colleagues found that after an initial reduction in total EDLOS (time in ED plus time in SSU), this reduction slowed in later years, indicating an increased reliance on the use of SSUs to meet target disposition times [69]. Their interview data indicated that transfer to a SSU was sometimes initiated without clinical justification in an effort to meet targets. Nevertheless, they acknowledged that from a patient perspective, time in the SSU is preferable to a longer EDLOS [69]. Jones et al. determined *a priori* quantitative changes that were deemed to be of clinical importance, regardless of statistical significance [48]. They reported clinically significant reductions in median IPLOS, median EDLOS, and access block hours [48]. Although there was no change in 2-day ED representations, they did report a clinically significant 1% increase in 30-day readmissions. Similar to Tenbensel and colleagues [69], Jones et al. reported an increase in use of SSUs, with < 5% of ED admissions to SSUs in 2009 (pre implementation) versus almost 13% in 2012 [48]. However, the latter study found statistically and clinically significant reductions in total EDLOS, which was greatest for admitted patients, indicating that the SSUs were not merely used to ‘stop the clock’.

#### Alternative admission policies

One American study explored the impact of a 14-bed monitored inpatient unit, staffed by the ED, on ED crowding [22]. Kelen and colleagues reported significant decreases in both rates of LWBS and hours of ambulance diversion [22]. Similarly, a Taiwanese study reported significant reductions in mean EDLOS for admitted patients after the introduction of a 14-bedded ‘high turnover’ unit, specifically used for ED admissions [51]. Utilising empty beds throughout the hospital in the out-of-hours period to accommodate non-specialist admissions to reduce EDLOS and avoid the need for inter-hospital transfers was trialled in one Dutch hospital [70]. The group reported no change in the EDLOS for patients eligible for admission to the new model, at a time when EDLOS for other patients increased significantly [70]. Providing the ED with extra assistance from hospital leaders and specialists during times of crowding in order to expedite patient disposition from the ED has been reported in two studies (capacity protocols) [41, 73]. The Korean study, which was investigating the long-term effects of the protocol, as it had been in place for six years, reported significant reductions in EDLOS [41]. Conversely, the American study, which reported on the effect of a relatively new intervention, reported a significant 34 minute increase in EDLOS on days when the full capacity protocol was operational [73]. They also reported a 92% significant decrease in hours of ambulance diversion related to the intervention [73].

## Discussion

### Consequences of crowding

A key finding of this review is that the consequences of ED crowding are well established. Reported consequences can be categorised as affecting patients, staff and the healthcare system, with some overlap. Some of the negative effects of crowding identified, such as adverse outcomes for patients, including treatment delays and increased mortality, were similar to those identified in Hoot’s review [8]. However, the previous review identified provider losses as a potential negative effect [8], a finding that was not replicated in the current review. Similarly, Hoot et al. reported impaired access to ED care, as measured by rates of LWBS and ambulance bypass, as potential consequences [8], whereas both of these measures were used as indicators of crowding in the current study.

The quality of the studies investigating consequences of crowding were variable, with only one high quality, prospective study included [19]. This was also the only study that did not find a link between crowding and the primary outcome measure, in this case increased inpatient mortality [19]. It did appear that the authors of some of the lower quality studies were determined to prove a negative consequence between ED crowding and their outcome of interest. For example, Kulstad and Kelly [81] concluded that crowding decreased the likelihood of timely treatment for acute myocardial infarction (AMI), when their study showed no relationship between crowding and time to first electrocardiogram or time to arrival in the cardiac catheterisation laboratory (CCL), which are the time stamps that ED staff have most influence over. Their study found a relationship between crowding and time to balloon inflation in the CCL, a delay that is presumably outside of the control of the ED [81].

Similarly, Hwang and colleagues [78] concluded that crowding is significantly associated with poorer pain management. Their study identified a negative association between crowding and time to assessment and documentation of pain, but no relationship to time to analgesic administration, that is, the outcome that affects patient care [78]. Rather than identifying negative outcomes for patients who present to crowded EDs, both of these studies could be taken to show the opposite. That is, that even when the ED is under stress, patients identified as having urgent clinical needs, such as those suffering from an AMI or being in severe pain, still receive appropriate, timely care. We acknowledge that the complexity of health services research provides challenges in terms of research design, often influencing investigators decisions’ to measure outcomes for which data is easily accessible. However, care needs to be taken when designing studies and interpreting results to ensure reported outcomes are robust and reflect the most appropriate measure of the phenomena under study.

### Solutions to crowding

Trialled and modelled solutions to ED crowding included providing alternative options to the ED for patient care, moving patients through the ED more quickly and expediting patients’ exit from the ED on completion of care. Many of these solutions were identified in the previous review [8], particularly the solutions aimed at resolving access block and providing alternative admission options. However, Hoot’s review identified many demand management strategies, including diverting patients to other forms of care and focussing on frequent visitors, which was the focus of only one, older study included in this review [32]. The demand management and patient diversion papers in the earlier review were all published more than twelve years ago, perhaps indicating the lack of long-term success of these initiatives at reducing ED crowding.

All studies included in this review evaluating solutions, with two exceptions [57, 58] reported significant improvements in measures of crowding related to the intervention, whether trialled or modelled. It should be noted that in Nagree’s study [58], that concluded that AHs GPs would have little impact on LAPs to EDs, the Sprivulis method [118] was used to calculate LAPs. This method consistently estimates a lower proportion of presentations as ‘GP-type’ than other methods [119, 120]. One Australian group reported a range of 15–69% of ED attendees as ‘GP-type’, depending on which of four definitions were used to calculate the proportion [119], with the Sprivulis method [118] producing the lowest percentage. Another Australian group [37] speculated that their finding of reduced LAPs to the ED following the opening of an AHs GP differed from Nagree’s findings because of the relative rural nature and therefore, lack of alternative options in the study locality, compared to the urban area studied by Nagree [58]. This finding is a clear indicator that a ‘one size fits all’ model to alleviate crowding is unlikely to be successful, as the causes of crowding are contextually specific to the environment in which the crowding occurs, and therefore requires solutions explicitly designed for that environment. The above also highlights the difficulties in comparing research outcomes when non-standardised definitions are employed as study outcome measures. This issue has been highlighted before [12, 13], with calls for a consensus on definitions for crowding, ‘GP-type’ presentations and LAPs to enable more accurate measuring and reporting of these issues.

#### Quality of solutions studies

The quality of the evidence evaluating solutions to ED crowding was higher than for the other two areas (causes and consequences) with 60% of the studies assessed as providing high or acceptable levels of evidence. Many input, throughput and output solutions, including WICs, providing earlier physician assessment on arrival to the ED, and providing alternative admission options during times of inpatient access block, have been found to have promising results. While POCT was trialled in five included studies, only two of these, both RCTs [26, 27], were assessed as providing high levels of evidence, suggesting more research needs to be undertaken in this area.

While the majority of the included papers, particularly those that looked at throughput initiatives, did not measure unintended ‘upstream’ effects of the interventions to reduce crowding, a number of the more recent ‘target’ papers did [48, 54, 62, 67]. The Australian papers reported increased in-hospital transfers, increased IPLOS [62], and a small clinically insignificant increase in ED representations within 48 hrs [67] as potentially negative clinical outcomes post-NEAT implementation. One NZ study reported a clinically important 1% increase in readmissions within 30 days [48]. The UK study found an unexpected increase in time to be seen by a clinician and reported that when EDLOS was adjusted for clustering by hospital, there was an increase in total time in the ED for admitted patients [54]. Overall, the ‘target’ studies provided acceptable levels of evidence of both improved processes and patient outcomes following their introduction, indicating that more research into the specific interventions undertaken to achieve targets, with an emphasis on understanding what worked, where and why, could go some way towards addressing ED crowding. Similarly, more recent studies have highlighted the positive effects of undertaking a whole-of-system approach, including involvement of system leaders and using available data for more effective communication as important strategies to reduce crowding [24, 67, 69].

Although one of the NZ ‘target’ studies [69] acknowledged some input strategies were implemented in at least one of their test sites, in the main ‘target’ studies focussed their reporting on throughput and output initiatives to address crowding. The two UK studies that reported reduced ED presentations following 7-day opening of GPs [43, 72], as well as the successes achieved after the opening of an AH GP clinic in a large regional centre [37], provide evidence to support further trials of increased access to primary care as a potential solution to crowding in areas where increased input has been identified as a causative factor.

#### Costs of solutions

A number of studies identified financial costs associated with the interventions [35, 43, 53, 69, 72], but did not provide any cost benefit analysis. One exception is an Australian study that calculated a $2,121 AUD per day saving to the ED after the introduction of a nurse navigator role [44]. Similarly, although not providing a comprehensive cost benefit analysis, Nagree et al. estimated that LAPs accounted for only 2.5% of total ED costs in the Perth metropolitan area, and therefore AH GPs were not a worthwhile investment if their aim was to reduce LAPs to the ED in a metropolitan setting [58]. Whittaker et al. acknowledged that while extended GP opening hours was seen to reduce patient-initiated ED referrals, extended opening hours may not produce a cost saving to the healthcare system [72].

### Causes of crowding

Surprisingly, the least number of studies included in this review investigated the causes of ED crowding. Causes included increases in types of ED presentations, limited access to primary care and access block for patients requiring admission. Access block, inadequate staffing and LAPs were also identified in Hoot’s [8] review as causes of crowding. However, a notable new identified cause in this review is the increase in presentations by patients with complex and chronic conditions, including the elderly, as a driver of ED crowding [29, 108, 114, 115]. This finding may indicate the emergence of a new driver of crowding, namely the elderly with multiple chronic conditions, and merits further investigation. The quality of the evidence investigating causes was mixed, with only seven (50%) studies assessed as being of acceptable quality, while the remainder were scored as low. Three of the higher quality studies identified access block as having a negative impact on ED crowding; however, all of these studies are more than ten years old [110–112]. The remaining four studies identified increased presentations by patients with chronic and complex care needs, including the elderly, and limited access to GPs, as causative factors of crowding [108, 109, 114, 117], adding further weight to the suggestion that increasing access to primary care may help to reduce crowding.

Fifteen years ago, Asplin [7] proposed in his conceptual model, that ED crowding could be partitioned into three interdependent components, input, throughput and output. Of the 14 studies that investigated the causes of ED crowding, only four identified a throughput issue, namely experience level of staff [113], shortages of staff within the ED [30, 31], and delays in test results and disposition decisions [20] as potential causative factors. However, of the 52 papers that trialled or modelled potential solutions to crowding, 31 (60%) involved improving patient throughput as a means of resolving the issue, with none of the interventions specifically targeted at improving staffing issues. This suggests a mismatch between the proven or accepted causes of crowding and the solutions developed and implemented to address the problem. There is general agreement that many of the causes and therefore solutions to crowding lie outside of the ED. However, our findings suggest that, as the most immediate effects of crowding are visible in the ED, ED clinicians have perhaps taken it upon themselves to change what they can influence to try to ameliorate the problem.

This review identified many new studies focussed on the ED crowding agenda. However, there is a paucity of research aimed at identifying the specific, contextual factors causing the phenomenon, with only eight new studies aimed at identifying causes published in the last ten years. The imbalance between the vast number of studies investigating the consequences and trialling solutions to ED crowding, versus the scarcity of studies aimed at identifying the causes, warrants attention. As stated by Asplin et al., ‘the development of valid and reliable measures of the factors **contributing** to ED crowding is the **first step** in developing a coherent research and policy agenda’ [7]. It appears that 15 years after this recommendation the ED research community is yet to thoroughly address that ‘first step’.

### Limitations

The literature search was limited to research published in English and in peer-reviewed journals. Potentially, a wider search strategy may have located a greater number of relevant studies; however, with the number of studies appraised and included, we feel this review provides a comprehensive analysis of the current research on ED crowding. Only seven of the included studies were assessed as being of high quality. This is an issue that has been highlighted before, with authors also acknowledging that it is difficult to critique complex and multi-faceted health service research using evaluation criteria designed for drug trials [121]. However, we elected to assess the quality of the evidence using traditionally accepted methods to enable the comparability of our results with previously published reviews. When allocating causes and solutions studies as related to either input, throughput or output, every effort was made to follow the original intentions of the study authors; however, this intention was not always clear.

### Conclusion

There is an abundance of research illustrating the negative consequences of ED crowding for patients, staff and the healthcare system. While many solutions have been trialled and modelled, with varying levels of success, there is a mismatch between the identified causes of crowding and the initiatives implemented in efforts to resolve the problem. More recent studies investigating the effects of timed disposition targets and extending GP opening hours have provided some promising results and warrant further investigation and evaluation, with a particular focus on which interventions worked in which contexts, relative to identified local causes of crowding. A significant finding of this review is the growing body of evidence suggesting elderly patients with complex, multi-morbid conditions represent an increasingly important driver of ED crowding. This review has highlighted the need for further, high quality research into the specific, contextual issues that lead to ED crowding and the tailoring of evidence-based solutions to address identified causes. There is agreement that the problem and therefore the solutions to ED crowding lie largely outside of the ED. Therefore, it is imperative that the whole of the system, including patients, are involved in identifying both the causes of and acceptable, sustainable solutions to ED crowding.

## Supporting information

S1 Table PRISMA checklist. (PDF)

S1 File Details of search strategy. (PDF)

S2 File Study protocol. (PDF)
