# Supplementary material for: Emergency department crowding: A systematic review of causes, consequences and solutions
Source: PLoS One. 2018 Aug 30;13(8):e0203316. doi: 10.1371/journal.pone.0203316 (PMC6117060; doi:10.1371/journal.pone.0203316)
Supplement: S1 File — (PDF) [file pone.0203316.s002.pdf]

S1 Details of search strategy

The following search strategy is an example of the strategy used on all databases. The search terms were selected based on commonly cited keywords in the literature around emergency department crowding.

Medline using Ovid (Jan 2000 to June 2018)

1. Accident and emergency **"OR"** ED **"OR"** Emergency Department **"OR"** Emergency service
2. Crowding **"OR"** Overcrowding **"OR"** Congestion **"OR"** Utilisation
3. #1 **"AND"** #2
4. Consequences **"OR"** Outcomes **"OR"** Harm **"OR"** Mortality **"OR"** Negative impact
5. Cause
6. Solution **"OR"** Strategies **"OR"** Intervention
7. #3 **"AND"** #4
8. #3 **"AND"** #5
9. #3 **"AND"** #6

LIMITS: English language AND peer-reviewed journal article AND Jan 1st 2000-Current

Table 1 Databases searched with dates of search and results.

Database	Date search	Results
Medline using Ovid	June 13th 2018	1019
Web of science	June 13th 2018	1289
Embase	June 13th 2018	1393
CINAHL	June 13th 2018	2065
