# Supplementary material for: Emergency department crowding: A systematic review of causes, consequences and solutions
Source: PLoS One. 2018 Aug 30;13(8):e0203316. doi: 10.1371/journal.pone.0203316 (PMC6117060; doi:10.1371/journal.pone.0203316)
Supplement: S2 File — (PDF) [file pone.0203316.s003.pdf]

The consequences and causes of, or solutions to, emergency department crowding: a systematic review protocol

Claire Morley, Maria Unwin, Gregory Peterson, Jim Stankovich, Leigh Kinsman

Citation

Claire Morley, Maria Unwin, Gregory Peterson, Jim Stankovich, Leigh Kinsman. The consequences and causes of, or solutions to, emergency department crowding: a systematic review protocol. PROSPERO 2017 CRD42017073439 Available from:
http://www.crd.york.ac.uk/PROSPERO/display_record.php?ID=CRD42017073439

Review question

What are the consequences of emergency department crowding?
What are the causes of emergency department crowding?
What solutions have been identified to combat emergency department crowding?

Searches

The search strategy aims to locate original research articles, published in peer reviewed journals, detailing the consequences or causes of, or the solutions to, emergency department (ED) crowding.
We will search the following electronic databases; MEDLINE, CINAHL, EMBASE and Web of Science.
The search strategy will include the following keywords; 'Emergency department', 'emergency service', 'accident and emergency', “AND” “OR” 'crowding', 'overcrowding', 'utilisation', 'congestion'
“AND” “OR” ‘consequences’, ‘outcomes’ ‘causes’ ‘strategies’, ‘solutions’,
'interventions'.
Studies published between January 2000 and June 2018 will be sought, and only studies published in the English language will be considered.
Studies undertaken in paediatric-only EDs will be excluded.

Types of study to be included

There will be no restriction to the types of studies considered for this review. Qualitative, quantitative and mixed-methods studies, that meet the inclusion criteria, will all be considered

Condition or domain being studied

Emergency department crowding, defined as occurring when, 'ED function is impeded primarily because the number of patients waiting to be seen, undergoing assessment and treatment, or waiting to leave exceeds the physical and/or staffing capacity of the ED' is the condition being studied.

Participants/population

This review will consider all studies that have investigated the consequences and/or causes of and/or solutions to ED crowding, in general EDs.
Studies undertaken in specialised EDs, such as paediatric EDs, will be excluded.

Intervention(s), exposure(s)

The exposure to be reviewed is ED crowding, as defined above.
There are many accepted measures of ED crowding, including ED length of stay, hours on ambulance bypass, and numbers of patients who leave the ED without being seen.
Studies that have incorporated a measure of ED crowding into their study design will be considered.
All studies that have investigated the consequences of ED crowding, or identified causes of ED crowding, or trialled or modelled solutions to ED crowding will be considered for inclusion.

Comparator(s)/control

This review will consider studies that compare outcomes for patients who present to crowded EDs versus those who present to non-crowded EDs.
This review will consider studies that compared different interventions to try to reduce ED crowding.
Studies without a comparator will also be considered.

Context

Studies undertaken in healthcare settings will be considered. This may be within the ED, but is also likely to include studies undertaken in primary care.

Primary outcome(s)

Primary outcome Q.1: outcomes that relate ED crowding to effects on patients, staff or the healthcare system.
Primary outcome Q.2: outcomes that show a cause and effect relationship between the identified cause and ED crowding.
Primary outcome Q.3: outcomes that show a positive, negative or null effect of a specified solution to ED crowding.

Secondary outcome(s)

None.

Data extraction (selection and coding)

Titles and/or abstracts of studies retrieved using the search strategy and those from additional sources will be screened by one review author to identify studies that potentially meet the inclusion criteria outlined above. The full texts of these potentially eligible studies will be retrieved and independently assessed for eligibility by two review team members. Any disagreements over the eligibility of particular studies will be resolved through discussion with a third reviewer.
A standardised form will be used to extract data from the included studies for assessment of study quality and evidence synthesis. Extracted information will include: study setting; study population; details of the intervention and control conditions; study methodology; outcomes and times of measurement.
Review authors will extract data in pairs, and any discrepancies will be identified and resolved through discussion with a third reviewer.

Risk of bias (quality) assessment

Two reviewers will independently assess the risk of bias of included studies using the SIGN critical appraisal tools. If there are any disagreements, a third reviewer will act as adjudicator.

Strategy for data synthesis

We will provide a narrative synthesis of the findings from the included studies. We will provide summaries of identified causes and consequences as well as solutions to ED crowding. We anticipate that the types of studies to be included are likely to be heterogeneous in their methodologies, making a meta-analysis of results unlikely.

Analysis of subgroups or subsets

None planned.

Contact details for further information

Claire Morley
claire.morley@utas.edu.au

Organisational affiliation of the review

University of Tasmania

Review team members and their organisational affiliations

Ms Claire Morley. University of Tasmania

Ms Maria Unwin. University of Tasmania and Tasmanian Health Service, North
Professor Gregory Peterson. University of Tasmania
Dr Jim Stankovich. University of Tasmania
Professor Leigh Kinsman. University of Tasmania and Tasmanian Health Service, North

Anticipated or actual start date

01 November 2016

Anticipated completion date

01 March 2018

Funding sources/sponsors

The primary author is in receipt of an Australian Government Research Training Program (RTP) Scholarship

Conflicts of interest

None known

Language

English

Country

Australia

Stage of review

Review_Completed_not_published

Subject index terms status

Subject indexing assigned by CRD

Subject index terms

Crowding; Delivery of Health Care; Emergency Medicine; Emergency Service, Hospital; Humans

Date of registration in PROSPERO

15 August 2017

Date of publication of this version

22 June 2018

Details of any existing review of the same topic by the same authors

Stage of review at time of this submission

Stage	Started	Completed
Preliminary searches	Yes	Yes
Piloting of the study selection process	Yes	Yes
Formal screening of search results against eligibility criteria	Yes	Yes
Data extraction	Yes	Yes
Risk of bias (quality) assessment	Yes	Yes
Data analysis	Yes	Yes

Versions

15 August 2017
05 June 2018
22 June 2018

PROSPERO
This information has been provided by the named contact for this review. CRD has accepted this information in good faith and registered the review in PROSPERO. CRD bears no responsibility or liability for the content of this registration record, any associated files or external websites.
